# IL‐7R‐Enriched Extracellular Vesicles From the Thymus Drive Colitis via Promoting Neutrophil Extracellular Trap Formation

**DOI:** 10.1002/advs.202520331

**Published:** 2026-07-06

**Authors:** Yao Liao, Yuheng Liu, Ruibing Yang, Zifeng Zhu, Junwei Wu, Dinghao Li, Jin Su, Yingxin He, Shiqi Luo, Feiyang Cao, Haiyi Deng, Lanmengxi Yang, Ling Zhong, Peiying Peng, Xinyi Wu, Chunmei Cai, Zhen Li, Yujin Wu, Shuofeng Zhu, Jie Wei, Yi Yang, Lifu Wang

**Affiliations:** ^1^ KingMed School of Laboratory Medicine The Affiliated Traditional Chinese Medicine Hospital Guangzhou Medical University Guangzhou China; ^2^ Engineering Technology Research Center of Intelligent Diagnosis for Infectious Diseases in Guangdong Province Guangzhou China; ^3^ Guangzhou Key Laboratory for Clinical Rapid Diagnosis and Early Warning of Infectious Diseases Guangzhou China; ^4^ Department of Parasitology of Zhongshan School of Medicine Sun Yat‐sen University Guangzhou China; ^5^ Institute of Virology Helmholtz Centre Munich‐German Research Centre for Environmental Health Munich Germany; ^6^ Chair for Preventions of Infectious Microbial Diseases Central Institute of Disease Prevention and School of Life Sciences Technical University of Munich Freising Germany; ^7^ The Department of Pathology The Second Affiliated Hospital of Guangzhou Medical University Guangzhou China; ^8^ Medical Department Xizang Minzu University Xianyang China; ^9^ The Department of Clinical Laboratory The Second Affiliated Hospital of Guangzhou Medical University Guangzhou China

**Keywords:** IL‐7R‐enriched extracellular vesicles, inflammatory bowel disease, neutrophil extracellular traps, thymus‐gut axis

## Abstract

Mounting evidence highlights the involvement of extra‐intestinal organs in inflammatory bowel disease (IBD) progression, yet the mechanisms underlying gut‐extraintestinal organ crosstalk remain poorly understood. Extracellular vesicles (EVs) serve as pivotal mediators of inter‐organ communication. Here, this study demonstrates that circulating EVs from colitis exacerbate colitis severity by inducing neutrophil extracellular trap (NET) formation. Inhibition of circulating EV secretion or NET formation alleviates colitis severity. Mechanistically, EVs enriched with interleukin‐7 receptor (IL‐7R) are identified as critical drivers of NET‐mediated colitis exacerbation. Downregulation of IL‐7R ameliorates colitis symptoms, whereas IL‐7R upregulation worsens disease progression. IL‐7R is found to induce NETs via the protein‐arginine deiminase type 4 (PAD4) pathway to aggravate colitis. Furthermore, this study establishes that thymus‐derived EVs are the primary source of IL‐7R‐enriched circulating EVs. During colitis, elevated circulating lipopolysaccharide (LPS) stimulates the thymus to release IL‐7R‐enriched EVs, which then migrate via the thymus‐gut axis and promote NET formation in colonic tissues. This study reveals a previously unrecognized thymus‐gut communication axis mediated by IL‐7R‐enriched EVs in IBD pathogenesis and provides an explanation for why IBD predominantly affects children and young adults. Targeting this axis may offer novel therapeutic strategies for IBD management.

## Graphical abstract

Inflammatory bowel disease (IBD) pathogenesis remains unclear. This study reveals that thymus‐originated IL‐7R‐enriched extracellular vesicles exacerbate colitis by promoting neutrophil extracellular traps via a thymus‐gut axis, uncovering an inter‐organ communication mechanism in inflammatory bowel disease pathogenesis. These findings provide a new therapeutic avenue for future IBD treatment strategies.

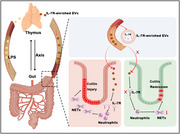

## 1 Introduction

Inflammatory bowel disease (IBD), encompassing ulcerative colitis (UC) and Crohn's disease (CD), is characterized by chronic intestinal inflammation, microbiome dysregulation, and impaired barrier function [1, 2]. Leukocyte infiltration at inflammatory sites, mediated by integrin‐endothelial receptor interactions, represents a hallmark of IBD pathology [3, 4]. The current treatments for IBD primarily include anti‐inflammatory agents, steroids, immunosuppressive drugs, and biological agents targeting inflammatory cytokines [5, 6]. However, these treatments primarily aim at symptom alleviation and do not address critical aspects such as mucosal epithelial healing, maintenance of barrier integrity, or correction of microbial imbalances within the gut [7]. The urgent need for mechanism‐based therapies underscores the importance of elucidating IBD pathogenesis [8].

Emerging evidence positions IBD as a systemic disorder involving multi‐organ crosstalk rather than a localized colonic disease. The brain‐gut axis influences IBD progression, with psychological interventions demonstrating therapeutic potential [9]. Similarly, the gut‐liver axis mediates bidirectional interactions through immune cells, microbiota metabolites, and bile acid regulation [10]. Clinical observations further reveal oral‐gut axis connections, where periodontitis and IBD exhibit epidemiological correlations [11]. Despite these advances, the molecular mediators orchestrating gut‐extraorgan communication remain elusive.

The thymus is essential for lifelong immunological tolerance and immunity. It exhibits distinctive epithelial complexity and undergoes age‐dependent involution [12], with age‐associated thymic involution linked to decreased immune function, increased susceptibility to infections, and a higher incidence of cancer and autoimmune diseases [13]. However, the underlying mechanisms remain unclear. James D. Lewis et al. reported that IBD primarily affects individuals aged 10–29 years [14], a period coinciding with thymic involution. In this study, we discovered that thymus‐derived extracellular vesicles (EVs) drive colitis via the thymus‐gut axis, potentially explaining why IBD predominantly affects children and young adults.

EVs have emerged as critical nanoscale messengers in systemic communication, transporting bioactive cargo (lipids, nucleic acids, proteins) across biological barriers. Remote tumor‐derived EVs and particles (EVPs) serve as crucial mediators of cancer‐induced hepatic reprogramming, which could be reversed by reducing tumor EVP secretion via depletion of Rab27a [15]. EVs from the lung pro‐thrombotic niche drive cancer‐associated thrombosis and metastasis via integrin beta 2 [16]. And adipose EVs regulate distant tissue gene expression via miRNAs [17].

Neutrophils, the most abundant cells in the human immune system, play a crucial role in innate immunity and perform their functions primarily through phagocytosis, degranulation, and the release of neutrophil extracellular traps (NETs) [18]. NETs are large, extracellular web‐like structures composed of cytoplasmic and granular proteins that are assembled on a scaffold of decondensed chromatin [19]. NETs trap, neutralize, and eliminate bacteria, fungi, viruses, and parasites, thereby preventing their spread [18]. However, disorders in NET formation can contribute to the development of immune‐related diseases. Patients with chronic perianal fistulizing CD have shown a significant increase in NETs [20]. An increase in NET formation was observed in mice with dextran sulfate sodium (DSS)‐induced colitis, and inhibiting this formation alleviated the clinical colitis index, intestinal inflammation, and barrier dysfunction [21, 22]. However, uncertainties persist regarding the precise mechanism underlying NET induction in IBD and their roles in IBD onset and progression.

The interleukin‐7 receptor (IL‐7R), essential for lymphoid development and immune homeostasis [23], shows clinical associations with anti‐TNF responsiveness in IBD [24]. However, its functional role in colitis pathogenesis is unknown. In this study, we identify circulating IL‐7R‐enriched EVs as novel mediators of thymus‐gut crosstalk that drive colitis progression via NET induction. These findings position IL‐7R‐enriched EVs as both diagnostic biomarkers and therapeutic targets in IBD.

## 2 Results

### 2.1 Circulating EVs From Colitis Induce NET Formation

**FIGURE 1:**
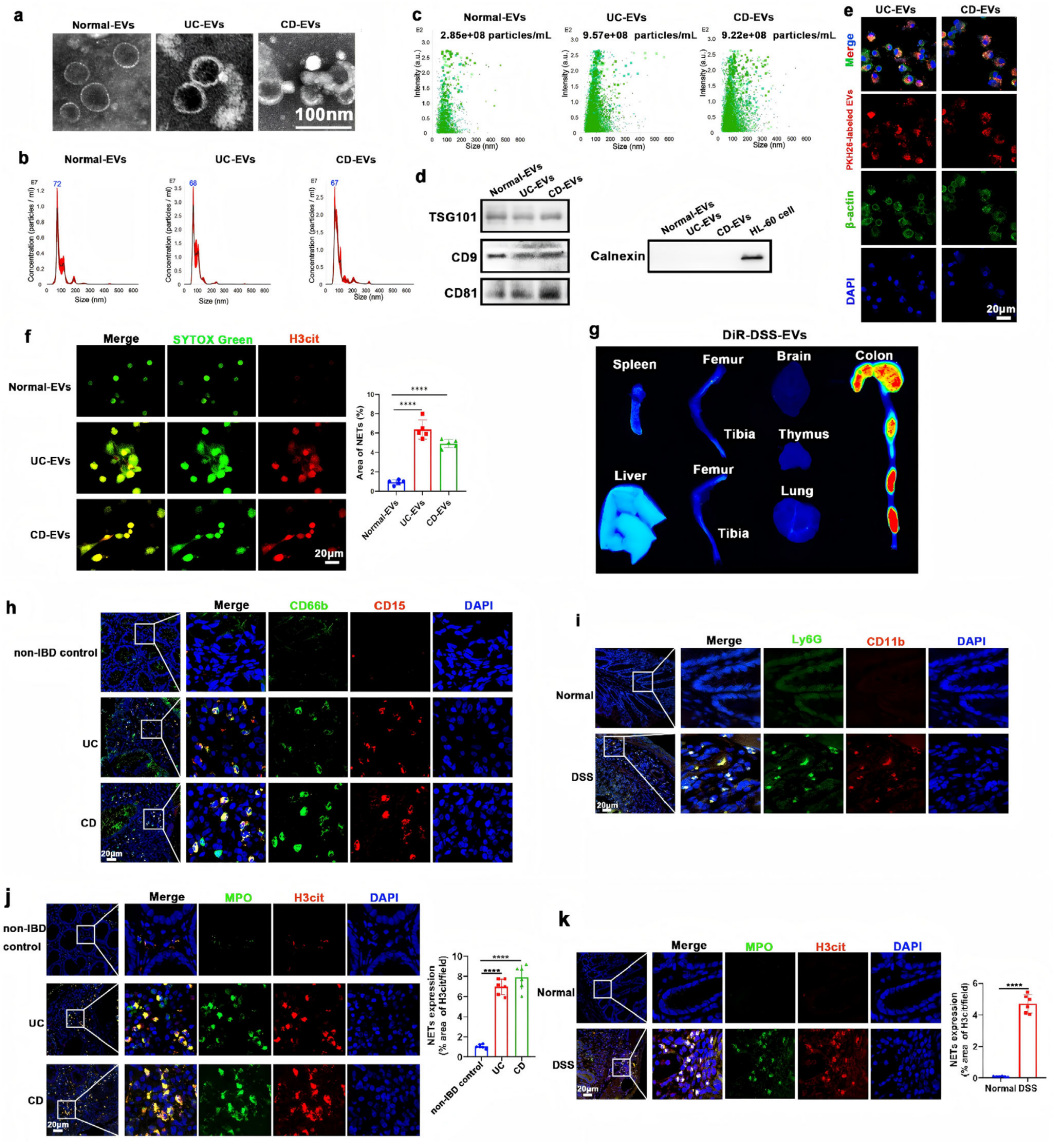
Circulating extracellular vesicles from colitis induce NET formation. (a) Plasma EVs from healthy volunteers (Normal‐EVs), UC patients (UC‐EVs), and CD patients (CD‐EVs) were analyzed using negative‐staining TEM. (b, c) EVs particles were investigated using nanoparticle tracking analysis. (d) Western blotting analysis of protein levels of TSG101, CD9, CD81, and Calnexin in EVs lysates, HL‐60 cell served as a positive control for the detection of Calnexin. (e) Neutrophils were incubated with PKH26‐labeled UC‐EVs and CD‐EVs, and EVs internalization was examined using laser scanning confocal microscopy. (f) Neutrophils were treated with Normal‐EVs, UC‐EVs, or CD‐EVs, and NET formation was assessed by SYTOX Green/H3cit co‐staining. (g) DiR‐labeled DSS‐EVs were intravenously injected into mice. *Ex vivo* imaging was performed to evaluate DiR‐DSS‐EV biodistribution in the colon, spleen, liver, femur, tibia, brain, thymus, and lung. (h) Immunofluorescence was used to analyze the level of neutrophils in colon tissues of IBD patients and non‐IBD controls. (i) Immunofluorescence analysis was performed to assess the level of neutrophils in the colon tissues of the experimental DSS‐induced colitis model. (j) Immunofluorescence analysis was conducted to evaluate the formation of NETs in colon tissues of IBD patients and non‐IBD controls. NET detection was based on H3cit and MPO co‐localization. (k) Immunofluorescence was used to assess the formation of NETs in the colon tissues of the experimental DSS‐induced colitis model. *n* = 6 mice per group; results are presented as the mean ± SD; ****p < 0.0001.

Circulating EVs serve as critical mediators of inter‐organ communication. To investigate whether circulating EVs mediate crosstalk between extraintestinal organs and the colon during IBD progression, we isolated plasma EVs from healthy volunteers (Normal‐EVs), UC patients (UC‐EVs), and CD patients (CD‐EVs). Transmission electron microscopy (TEM) and nanoparticle tracking analysis (NTA) confirmed the characteristic cup‐shaped morphology of the EVs, with a vesicle size distribution between 30 and 200 nm, consistent with the ideal size for EVs (Figure 1a–c). Notably, UC (9.57e + 08 particles/mL) and CD (9.22e + 08 particles/mL) patients exhibited significantly higher plasma EV concentrations than healthy controls (2.85e + 08 particles/mL) (Figure 1c). To verify EV identity and purity, we conducted Western blotting analysis for key EV protein markers. The EVs showed consistent expression of the hallmark EV markers TSG101, CD81, and CD9 (Figure 1d). In contrast, Calnexin, an endoplasmic reticulum marker and negative control for EVs, was absent, confirming that the EV preparations were free from cellular contamination. (Figure 1d). Dysregulated mucosal immune responses are at the heart of colitis pathogenesis characterized by increased local accumulation of immune cells, most notably neutrophils, which are associated with architectural distortion of tissue, crypt destruction, and crypt abscess formation [25]. We observed that plasma EVs from UC and CD patients were internalized by neutrophils and potently induced NET formation (Figure 1e,f; Figure S1).

To determine whether circulating EVs migrate to the colon to drive NETosis, we intravenously injected DiR‐labeled EVs derived from DSS‐induced colitis mice (DiR‐DSS‐EVs) and tracked their biodistribution. DiR‐DSS‐EVs accumulated in the colon, liver, and spleen 24 h post‐injection (Figure 1g). In parallel, EVs were also administered via intraperitoneal injection and intragastric gavage, and they were consistently found to accumulate in the colon (Figure S2).

Further analysis revealed substantial neutrophil infiltration and NET formation in colonic tissues from UC patients, CD patients, and DSS‐induced colitis mice (Figure 1h–k). These findings suggest that circulating EVs reach the colon and trigger NET formation.

### 2.2 Inhibition of EV Secretion Downregulates Colonic NET Formation and Alleviates Experimental Colitis in Mice

**FIGURE 2:**
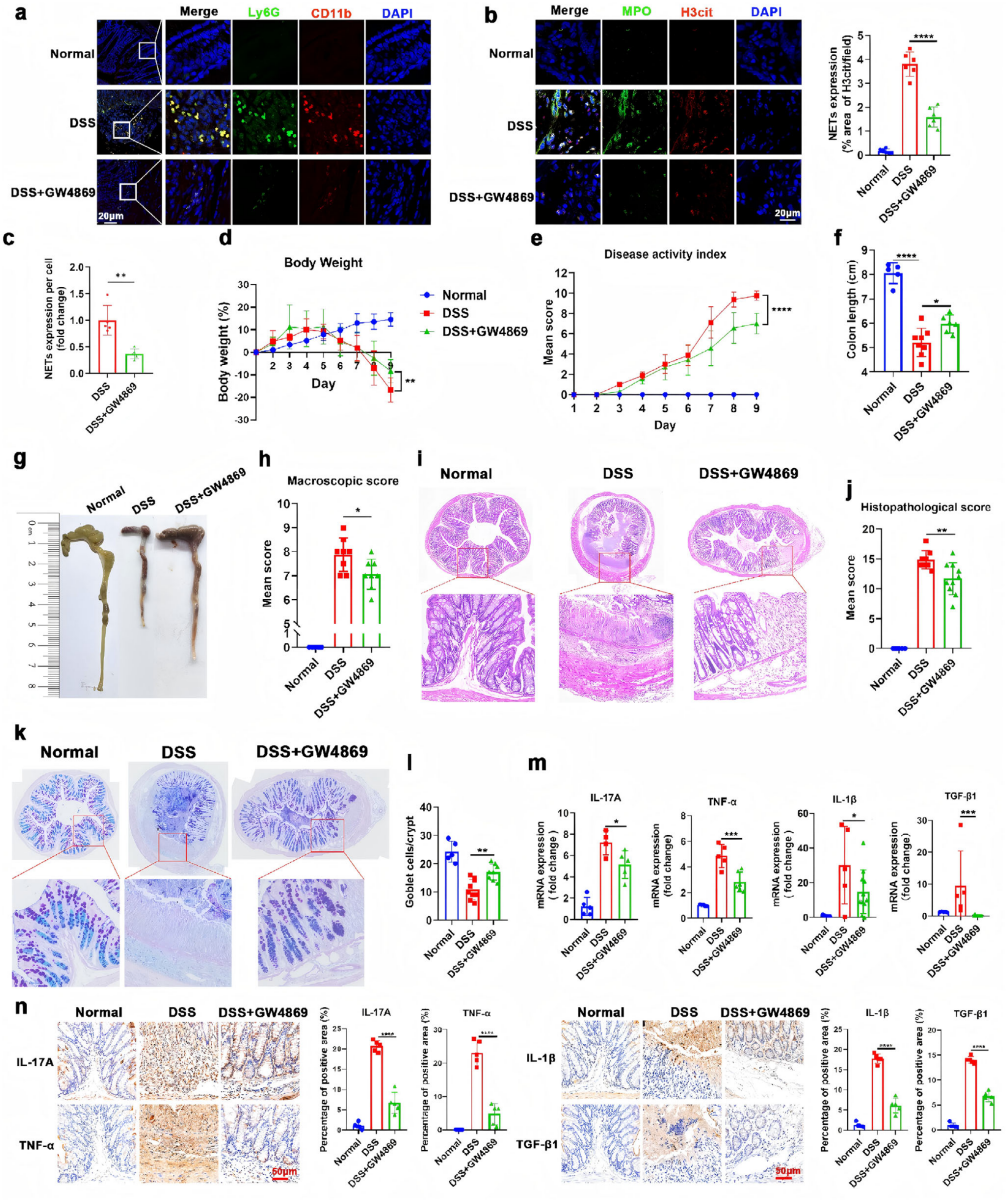
Inhibition of EV secretion downregulates colonic NET formation and alleviates experimental colitis in mice. (a) Analysis of neutrophil levels in colon tissues was conducted using immunofluorescence. (b, c) NET detection was based on H3cit and MPO co‐localization, and the area of overall NET formation as well as the NET area formed by single cells were further analyzed. (d) The body weight loss observed during the colitis course relative to Day 1 (which was set as 0%). (e) Changes in DAI. (f) Mice were sacrificed on Day 9, their colons removed, and the lengths measured and recorded. (g) Characterization of the macroscopic appearance of the colon. (h) Mean macroscopic scores of the colon for each group. (i) Histopathological changes in colon tissue samples were examined using H&E staining. (j) Histopathological scores were determined for the colon tissue samples. (k, l) Goblet cell depletion in the colon was analyzed using AB‐PAS staining (k), and goblet cells were quantified (l). (m) Relative mRNA expression levels of inflammatory cytokines in colon tissue were quantified using qRT‐PCR. (n) Protein expression of IL‐17A, TNF‐α, IL‐1β, and TGF‐β1 in colon tissue (immunohistochemistry). *n* = 5–9 mice per group; results are presented as the mean ± SD; *p < 0.05, **p < 0.01, ***p < 0.001, ****p < 0.0001.

To investigate the role of circulating EVs in promoting NET formation during colitis, we inhibited EV release using GW4869 (Figure S3), a selective EV biogenesis inhibitor [26]. EV blockade significantly reduced neutrophil recruitment and NET formation in the colon compared to DSS‐treated controls (Figure 2a–c). NETosis is regulated by reactive oxygen species (ROS)‐associated signaling pathways involving IL‐1 receptor‐associated kinase (IRAK4), receptor‐interacting serine/threonine‐protein kinase 1/3 (RIPK1/3), phosphatidylinositol 3‐kinase (PI3K), mechanistic Target of Rapamycin (mTOR), mitogen‐activated protein kinase (MAPK), and protein‐arginine deiminase type 4 (PAD4) [18]. Activation of RIPK3 and mTOR has been shown to upregulate PAD4, thereby facilitating NET formation [18]. We found that inhibition of EV release reduced the expression levels of IRAK4, RIPK1, RIPK3, PI3K, mTOR, MAPK, and PAD4 (Figure S4), suggesting circulating EV‐mediated regulation of NETosis pathways.

EV inhibition attenuated colitis severity, as evidenced by reduced weight loss (Figure 2d), lower disease activity index (DAI) (Figure 2e), longer colon lengths (Figure 2f,g), and lower mean macroscopic colon scores (Figure 2h) compared to the DSS group. Furthermore, histological analyses revealed that treatment with GW4869 significantly reduced disruption of colonic architecture compared to the DSS group (Figure 2i), with the histological scores of the colitis mice subjected to EV inhibition being significantly lower than those of the DSS group (Figure 2j). Moreover, goblet cell depletion induced by DSS was mitigated after GW4869 treatment (Figure 2k,l). EV inhibition resulted in significant downregulation of both mRNA and protein levels of the proinflammatory factors IL‐17A, TNF‐α, IL‐1β, and TGF‐β1 (Figure 2m,n). To further clarify whether the reduction in NET formation after GW4869 treatment was due to the inhibition of EV secretion or a direct inhibitory effect of GW4869 on neutrophils, we stimulated neutrophils with sufficient UC‐EVs, CD‐EVs, and PMA in the presence of GW4869. We found that GW4869 did not decrease NET formation induced by UC‐EVs, CD‐EVs, or PMA (Figure S5). Collectively, these results demonstrate that blocking EV secretion mitigates colonic NET formation and ameliorates experimental colitis.

### 2.3 Promotion of NET Formation Is the Key Mechanism by Which Circulating Extracellular Vesicles Exacerbate Colitis

To investigate whether the promotion of NET formation is the critical mechanism through which circulating extracellular vesicles aggravate colitis, we first reduced colonic neutrophils in DSS‐induced colitis mice by intraperitoneal injection of an anti‐Ly‐6G antibody to indirectly suppress NET formation (Figure S6a,b). Notably, the formation of NETs in the colon was significantly attenuated following treatment with an anti‐Ly‐6G antibody (Figure S7).

**FIGURE 3:**
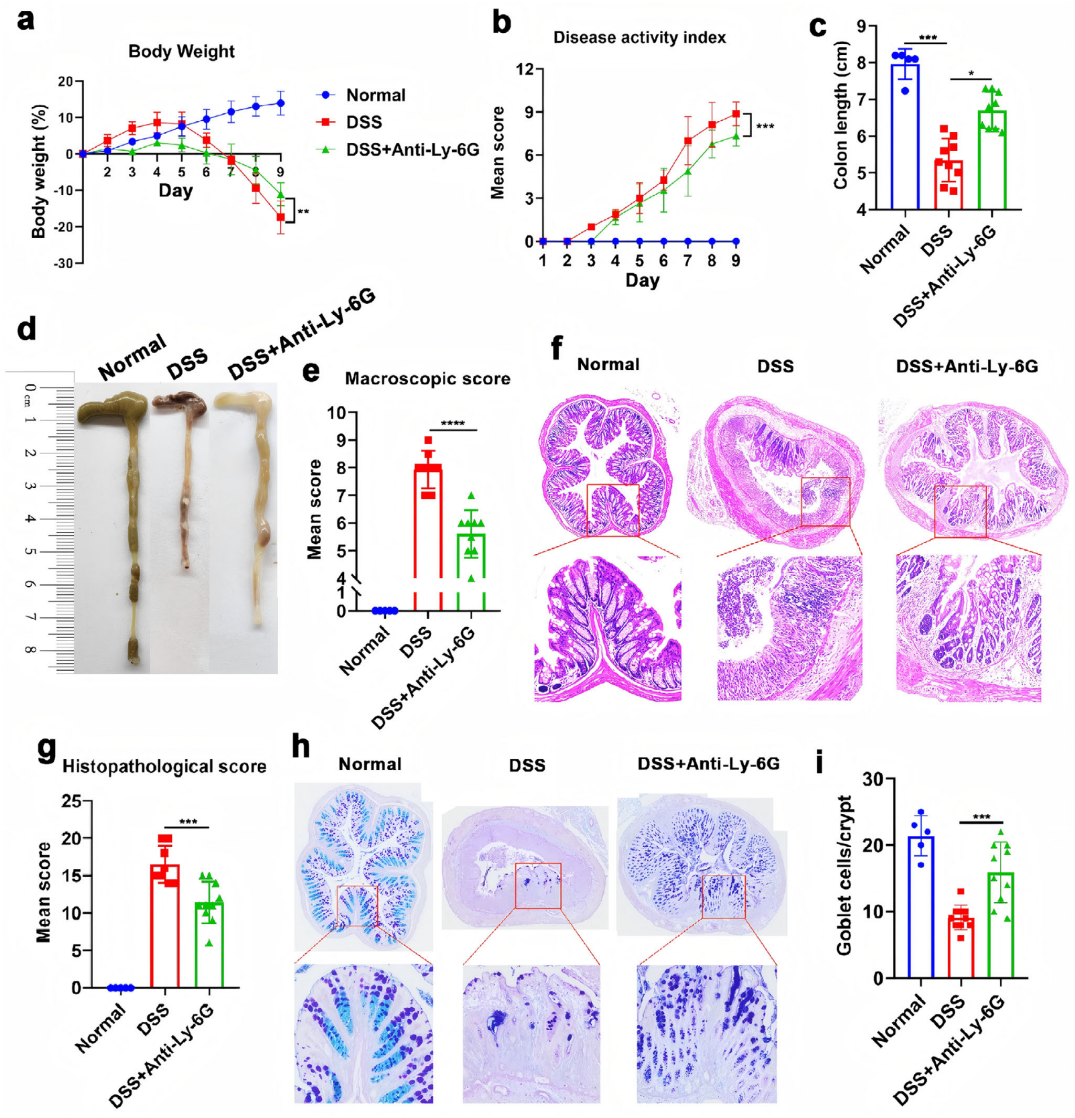
Neutrophil depletion alleviates colitis. (a) The body weight loss observed during the colitis course relative to Day 1 (which was set as 0%). (b) Changes in the DAI. (c) On Day 9, the mice were euthanized, their colons removed, and the lengths measured and recorded. (d) Macroscopic appearance of the colon, as represented by the colon with the mean colon length and typical injury findings. (e) Mean colon macroscopic scores for each group were evaluated. (f) Histopathological changes in the colon tissue samples were examined using H&E staining. (g) Histopathological scores for the colon tissue samples were determined. (h, i) Goblet cell depletion assessed by AB‐PAS staining (h) and quantified by goblet cell counting (i). n = 5‐10 mice per group; results are presented as the mean ± SD; **p < 0.01, ***p < 0.001, ****p < 0.0001.

Compared to the DSS group, the mice in the DSS + anti‐Ly‐6G group showed reduced body weight loss from Day 8 (Figure 3a), decreased DAI (Figure 3b), increased colon lengths (Figure 3c,d), and lower mean macroscopic colon scores (Figure 3e). Moreover, histological analysis revealed that treatment with anti‐Ly‐6G antibody significantly reduced disruption of colonic architecture compared to the DSS group (Figure 3f), with histological scores of the treated mice being significantly lower (Figure 3g). Additionally, goblet cell depletion induced by DSS was ameliorated following anti‐Ly‐6G antibody treatment (Figure 3h,i). Furthermore, treatment with anti‐Ly‐6G antibody resulted in significant downregulation of the levels of the proinflammatory factors TNF‐α, iNOS, and Arg‐1 (Figure S8).

**FIGURE 4:**
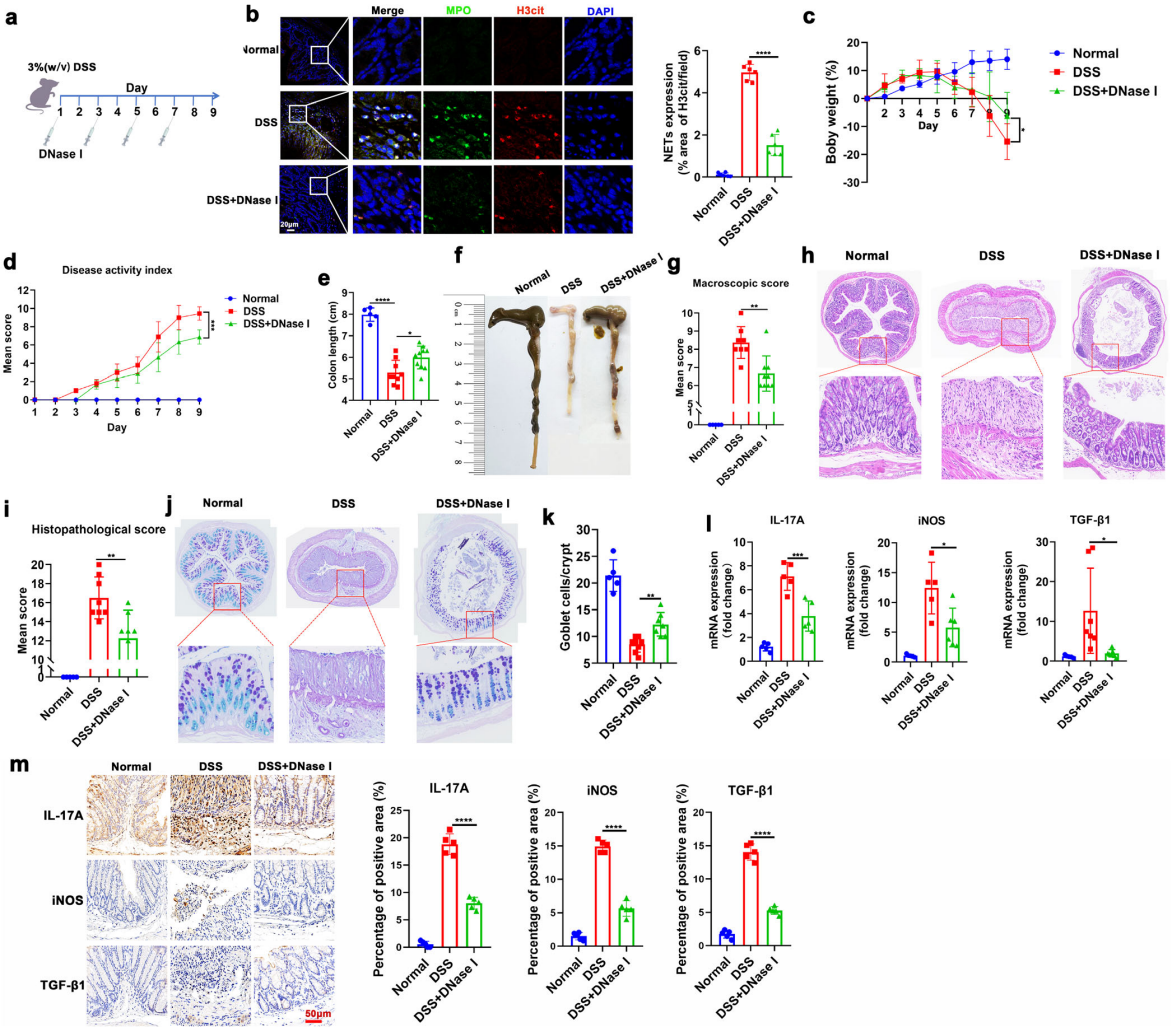
NET formation mediates circulating extracellular vesicle‐aggravated colitis. (a, b) Intraperitoneal injections of DNase I were administered on days 1, 3, 5, and 7 to reduce the formation of NETs in DSS‐induced colitis mice (DSS + DNase I). Immunofluorescence was used to analyze the formation of NETs in colon tissues. (c) Body weight loss during colitis course relative to Day 1 (set as 0%). (d) Changes in the DAI. (e) On Day 9, mice were euthanized, their colons removed, and the lengths measured and recorded. (f) Characterization of the macroscopic appearance of the colon. (g) Mean macroscopic scores of the colon per group. (h) Histopathological changes in the colon tissue samples were examined using H&E staining. (i) Histopathological scoring for colon tissue samples. (j, k) AB‐PAS staining was used to assess goblet cell depletion in the colon (j), and goblet cells were quantified (k). (l) Relative mRNA expression levels of IL‐17A, iNOS, and TGF‐β1 in colon tissue were quantified by qRT‐PCR. (m) IL‐17A, iNOS, and TGF‐β1 protein expression in colon tissue was detected by immunohistochemistry. *n* = 5–10 mice per group; results are presented as the mean ± SD; *p < 0.05, **p < 0.01, ***p < 0.001, ****p < 0.0001.

Neutrophils exert their functions through multiple mechanisms, including phagocytosis, degranulation, and NET formation. To specifically confirm that NET induction—rather than modulation of other neutrophil functions—is the key mechanism by which circulating extracellular vesicles exacerbate colitis, we administered intraperitoneal DNase I to degrade NETs in DSS‐induced colitis mice (DSS + DNase I group) (Figure 4a,b). Compared to the DSS group, the mice in the DSS + DNase I group showed reduced body weight loss from Day 8 (Figure 4c), lower DAI scores (Figure 4d), increased colon lengths (Figure 4e,f), and lower mean macroscopic colon scores (Figure 4g). Histological analysis further revealed that DNase I treatment significantly preserved colonic architecture compared to the DSS group (Figure 4h), accompanied by significantly lower histopathological scores (Figure 4i). Additionally, goblet cell depletion induced by DSS was ameliorated following DNase I treatment (Figure 4j,k). DNase I treatment led to significant downregulation of both mRNA and protein levels of proinflammatory factors, including IL‐17A, iNOS, and TGF‐β1 (Figure 4l,m). Collectively, these findings demonstrate that circulating extracellular vesicles exacerbate experimental colitis primarily through NET induction.

### 2.4 Plasma‐Derived EVs of Colitis Are Enriched With IL‐7R and Induce Colonic NET Formation Through IL‐7R

**FIGURE 5:**
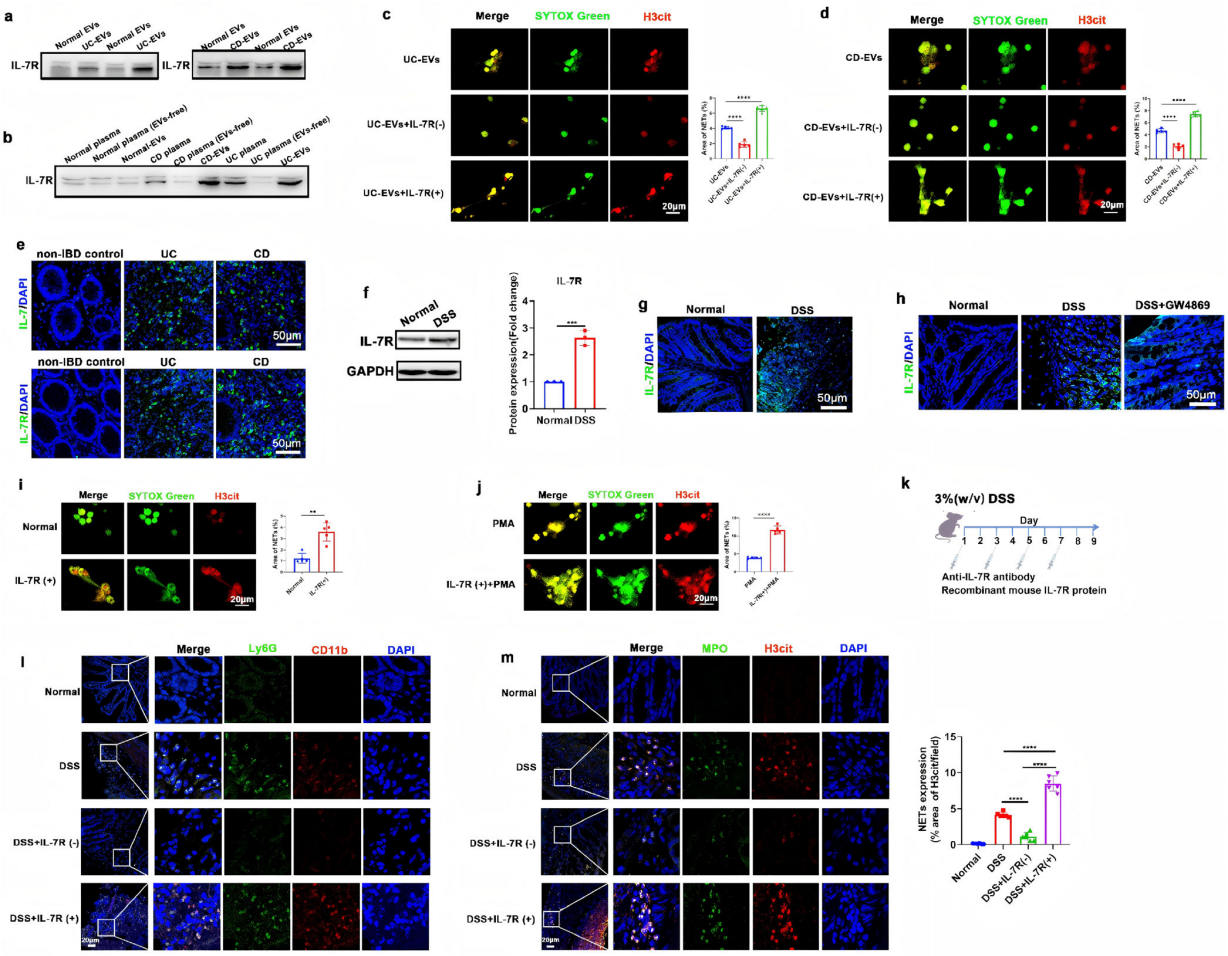
Plasma‐derived EVs of colitis are enriched with IL‐7R and induce colonic NET formation through IL‐7R. (a) IL‐7R levels in EVs were detected using western blotting. (b) The levels of IL‐7R in plasma, EVs‐free plasma, and EVs were detected by western blotting. (c) Neutrophils were treated with UC‐EVs, UC‐EVs + anti‐IL‐7R antibody, or UC‐EVs + recombinant mouse IL‐7R protein, and NET formation was assessed by SYTOX Green/H3cit co‐staining. (d) Neutrophils were treated with CD‐EVs, CD‐EVs + anti‐IL‐7R antibody, or CD‐EVs + recombinant mouse IL‐7R protein, and NET formation was assessed by SYTOX Green/H3cit co‐staining. (e) Immunofluorescence analyses were performed to evaluate IL‐7 and IL‐7R expression in colon tissues from IBD patients and non‐IBD controls. (f, g) Western blotting and immunofluorescence analyses were used to assess the levels of IL‐7R in the colon tissues of the experimental DSS‐induced colitis model. (h) Intraperitoneal injections of GW4869 were administered on days 1, 3, 5, and 7 to inhibit EVs release in DSS‐induced colitis mice, and immunofluorescence analysis was performed to examine IL‐7R expression in colon tissues. (i) Immunofluorescence analysis demonstrating that IL‐7R promotes the formation of NETs. (j) Immunofluorescence showing that IL‐7R can promote the formation of PMA‐induced NETs. (k) Intraperitoneal injections of anti‐IL‐7R antibody (DSS + IL‐7R (+)) and recombinant mouse IL‐7R protein (DSS + IL‐7R (‐)) were administered on days 1, 3, 5, and 7 to modulate IL‐7R expression in DSS‐induced colitis mice. (l) On Day 9, the mice were sacrificed, and the level of neutrophils in the colon tissues was measured by immunofluorescence. (m) Immunofluorescence was used to analyze the formation of NETs in colon tissues. *n* = 5–10 mice per group; results are presented as the mean ± SD; ***p < 0.001, ****p < 0.0001.

IL‐7R signaling plays an important role in the development and progression of autoimmune disease, and abnormal homing activity and steroid resistance caused by IL‐7R signaling may worsen prognosis [27]. We found that the level of IL‐7R in plasma EVs of UC and CD patients was significantly higher than that of healthy volunteers (Figure 5a). Furthermore, comparing IL‐7R levels in plasma, EVs‐free plasma, and EVs revealed significantly higher levels of IL‐7R in both plasma and EVs of UC and CD patients (Figure 5b). Notably, our results indicated that plasma IL‐7R levels were elevated in UC and CD patients, with IL‐7R mainly enriched in plasma EVs (Figure 5b).

To investigate the role of IL‐7R in UC and CD EVs‐induced NET formation, we treated neutrophils with UC and CD EVs in conjunction with anti‐IL‐7R antibody or recombinant mouse IL‐7R protein to downregulate or upregulate IL‐7R levels in EVs. IL‐7R inhibition significantly decreased UC‐EVs‐mediated induction of NET formation, while IL‐7R upregulation increased NET formation (Figure 5c, Figure S9a). Similar effects were observed with CD‐EVs (Figure 5d, Figure S9b). These findings suggest that plasma‐derived EVs from IBD patients carry IL‐7R in high abundance and induce NET formation through IL‐7R.

IL‐7/IL‐7R signaling has been implicated in the pathogenesis of autoimmune diseases [23]. To determine whether circulating EVs deliver IL‐7R to the colon to exert functional effects, immunofluorescence analysis was performed to assess IL‐7 and IL‐7R levels in colon tissues from IBD patients and non‐IBD controls. IL‐7 and IL‐7R levels were significantly elevated in the colonic mucosa of UC and CD patients compared to non‐IBD controls (Figure 5e). Subsequently, IL‐7R levels were evaluated in a dextran sulfate sodium (DSS)‐induced experimental colitis model. Western blotting and immunofluorescence analyses confirmed upregulated IL‐7R levels in colonic tissues of DSS‐induced colitis mice (Figure 5f,g). Strikingly, inhibition of EV secretion significantly reduced colonic IL‐7R protein levels (Figure 5h). These findings indicate that a substantial portion of colonic IL‐7R originates from circulating EVs.

To investigate whether colonic IL‐7R is critical for NET induction, in vitro experiments revealed that IL‐7R stimulation directly promoted NET formation (Figure 5i, Figure S10a) and enhanced phorbol 12‐myristate 13‐acetate (PMA)‐induced NETs generation (Figure 5j, Figure S10b). For in vivo validation, anti‐IL‐7R antibody or recombinant mouse IL‐7R protein was administered intraperitoneally to downregulate (DSS + IL‐7R (‐)) or upregulate (DSS + IL‐7R (+)) colonic IL‐7R levels in DSS‐induced colitis mice (Figure 5k). As shown in Figure S10c, compared with the DSS group, the level of IL‐7R in colon tissues was significantly downregulated after anti‐IL‐7R antibody treatment, whereas IL‐7R in colon tissues was significantly increased following recombinant mouse IL‐7R protein treatment. IL‐7R downregulation markedly attenuated neutrophil recruitment and NET formation in the colon, while IL‐7R upregulation exacerbated these effects (Figure 5l,m). Furthermore, IL‐7R inhibition reduced expression of key NETs‐associated molecules (RIPK3, mTOR, and PAD4), whereas IL‐7R overexpression enhanced their levels (Figure S11). These results confirm that colonic IL‐7R is pivotal for NET induction.

Collectively, these findings demonstrate that plasma‐derived EVs in colitis are enriched with IL‐7R and drive colonic NET formation via IL‐7R.

### 2.5 IL‐7R Exacerbates the Severity of Experimental DSS‐Induced Colitis

**FIGURE 6:**
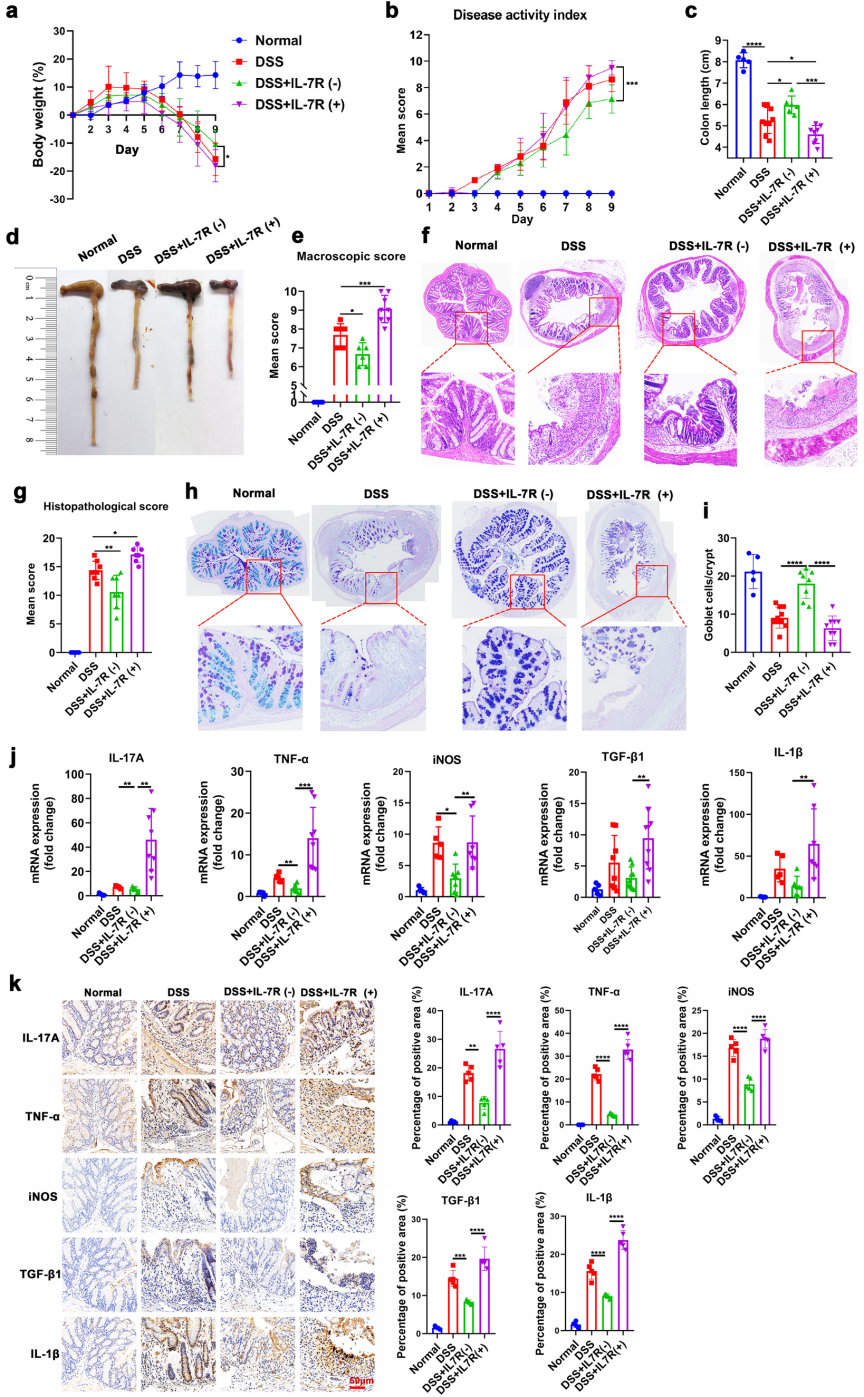
IL‐7R exacerbates the severity of experimental DSS‐induced colitis. (a) Mice with DSS‐induced colitis received either anti‐IL‐7R antibodies or recombinant mouse IL‐7R to modulate IL‐7R expression, with body weight changes monitored over time (Day 1 was set as 0%). (b) Changes in the DAI, which were assessed based on diarrhea, bleeding, and body weight loss. (c) Colon length of mice with different treatments. (d) Macroscopic appearance of the colon, as represented by the colon with the mean colon length and typical injury findings. (e) Mean colon macroscopic scores were evaluated for each group. (f) Histopathological changes in the colon tissue samples were examined using H&E staining. (g) Histopathological scores for the colon tissue samples were determined. (h, i) Goblet cell depletion in the colon was assessed using AB‐PAS staining (h) and quantified by counting goblet cells (i). (j) Relative mRNA expression levels of the inflammatory cytokines in colon tissue were quantified by qRT‐PCR. (k) IL‐17A, TNF‐α, iNOS, TGF‐β1, and IL‐1β protein expression in colon tissue was detected by immunohistochemistry. *n* = 5–10 mice per group; results are presented as the mean ± SD; *p < 0.05, **p < 0.01, ***p < 0.001, ****p < 0.0001.

To determine whether circulating EVs delivering IL‐7R to the colon aggravate colitis by inducing NETs, we assessed disease severity in DSS + IL‐7R (‐) and DSS + IL‐7R (+) mice. Compared to the DSS group, the DSS + IL‐7R (‐) group mice showed significantly reduced body weight loss beginning on Day 8, while the DSS + IL‐7R (+) group mice experienced aggravated weight loss (Figure 6a). The DAI of the DSS + IL‐7R (‐) group mice was lower than that of DSS controls from Day 7, whereas DSS + IL‐7R (+) mice displayed higher DAI scores (Figure 6b). Colon lengths in the DSS + IL‐7R (‐) group were longer than those in the DSS group, while DSS + IL‐7R (+) mice had shorter colons (Figure 6c,d). Macroscopic colon damage scores were significantly reduced in DSS + IL‐7R (‐) mice but elevated in DSS + IL‐7R (+) mice (Figure 6e).

Anti‐IL‐7R antibody treatment alleviated DSS‐induced colonic architectural disruption, whereas recombinant IL‐7R protein worsened it (Figure 6f). Consistently, histological scores were lower in anti‐IL‐7R‐treated mice but higher in IL‐7R‐overexpressing mice compared to DSS controls (Figure 6g). DSS‐induced goblet cell depletion was mitigated by IL‐7R inhibition but exacerbated by IL‐7R upregulation (Figure 6h,i). Proinflammatory cytokine levels (IL‐17A, TNF‐α, iNOS, TGF‐β1, and IL‐1β) were reduced in anti‐IL‐7R‐treated mice but elevated in IL‐7R‐overexpressing mice (Figure 6j,k). These results highlight a strong correlation between IL‐7R and IBD severity.

### 2.6 IL‐7R Induces NETs via the PAD4 Pathway to Aggravate Colitis

**FIGURE 7:**
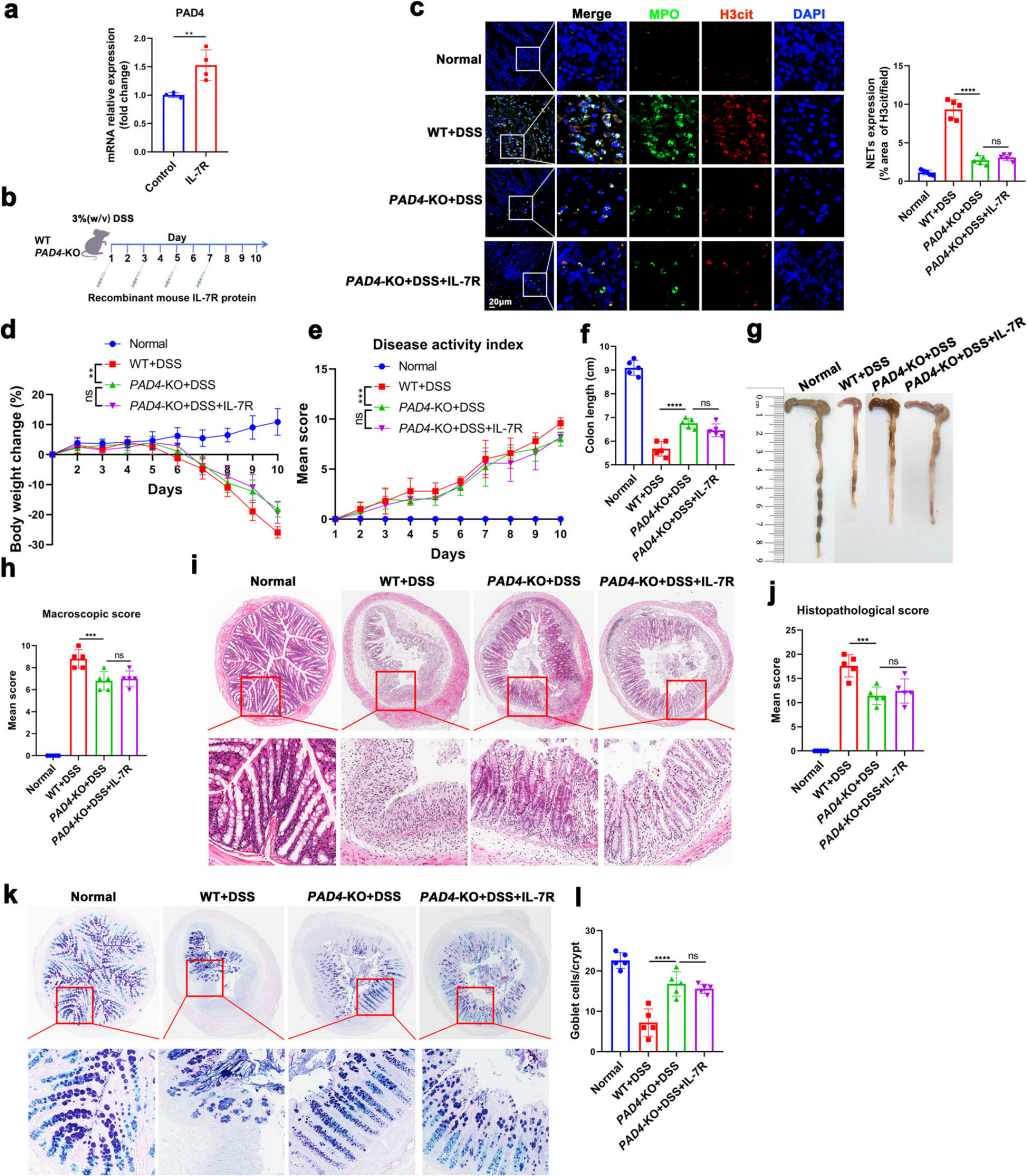
IL‐7R aggravates colitis by inducing NETs via the PAD4 pathway. (a) Relative mRNA expression level of PAD4 in neutrophils stimulated with IL‐7R (qRT‐PCR). (b) Schematic of IL‐7R protein administration to WT and *PAD4*‐KO DSS‐induced colitis mice. (c) Immunofluorescence analysis and quantification of NET formation in colon tissues. (d) Percentage change in body weight relative to Day 1. (e) Changes in the DAI. (f) Colon lengths measured on Day 10. (g) Characterization of the macroscopic appearance of the colon. (h) Mean macroscopic colon damage scores. (i) Representative H&E‐stained sections of colon tissue. (j) Histopathological scores. (k, l) Representative AB‐PAS stained sections (k) and quantification of goblet cells (l). *n* = 5 mice per group; results are presented as the mean ± SD; **p < 0.01, ***p < 0.001, ****p < 0.0001; ns: non‐significance.

In autoimmune diseases, the PAD4 pathway is activated and is a key factor in dysregulated NET formation [28]. We found that inhibition of EV release reduced, while IL‐7R overexpression enhanced, the expression level of PAD4 in the colons of colitis mice (Figures S4 and S11). Furthermore, IL‐7R stimulation significantly upregulated PAD4 expression in neutrophils (Figure 7a). To investigate whether IL‐7R induces NETs and aggravates colitis through the PAD4 pathway, we treated *Padi4* gene knockout (*PAD4*‐KO) colitis mice with IL‐7R protein (Figure 7b). We found that NET formation was significantly downregulated in the colons of *PAD4*‐KO colitis mice compared to wild‐type (WT) colitis mice. Moreover, IL‐7R treatment failed to increase NET formation in *PAD4*‐KO mice (Figure 7c), indicating that the PAD4 pathway is essential for IL‐7R‐induced NETosis.

Compared to WT colitis mice, *PAD4*‐KO colitis mice showed reduced body weight loss (Figure 7d), lower DAI scores (Figure 7e), increased colon lengths (Figure 7f,g), and lower macroscopic colon damage scores (Figure 7h). Histological analysis revealed that *PAD4*‐KO colitis mice had significantly preserved colonic architecture and lower histopathological scores (Figure 7i,j). Additionally, DSS‐induced goblet cell depletion was ameliorated in *PAD4*‐KO mice (Figure 7k,l). Importantly, IL‐7R treatment did not significantly alter any of these disease parameters in *PAD4*‐KO mice (Figure 7d–l). These findings indicate that IL‐7R aggravates colitis by inducing NETs via the PAD4 pathway.

### 2.7 The Thymus Is the Primary Source of Circulating IL‐7R‐Enriched EVs

**FIGURE 8:**
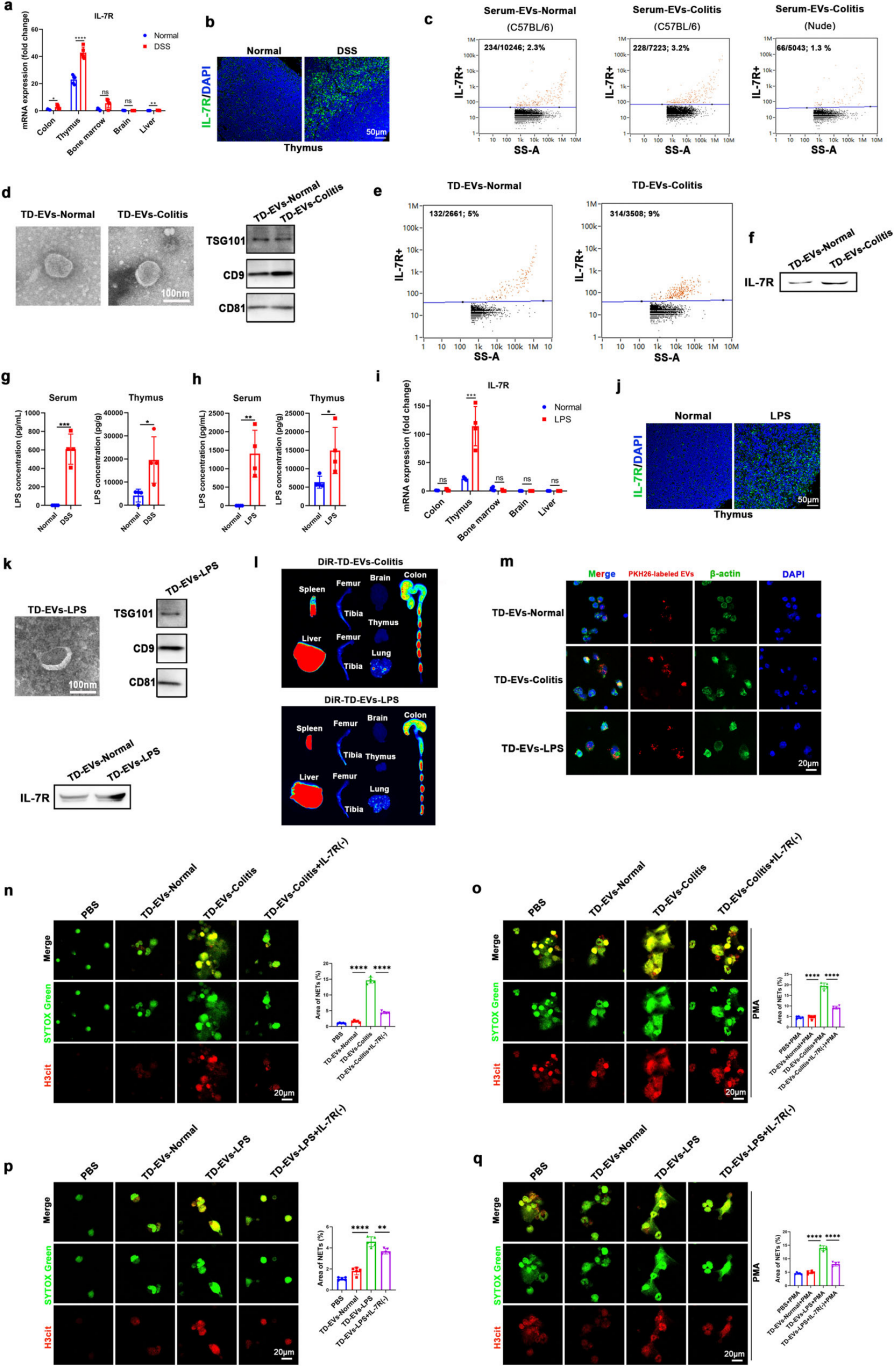
The thymus is the primary source of circulating IL‐7R‐enriched EVs. (a) qRT‐PCR analysis of IL‐7R expression in colon, thymus, bone marrow, brain, and liver tissues of mice. (b) Immunofluorescence evaluation of IL‐7R expression in the thymus. (c) Nanoflow cytometry analysis of IL‐7R levels in serum EVs. (d) TD‐EVs‐Normal and TD‐EVs‐Colitis were analyzed using negative‐staining TEM and western blotting detection of TSG101, CD9, and CD81. (e) Nanoflow cytometry analysis of IL‐7R levels in TD‐EVs‐Normal and TD‐EVs‐Colitis. (f) Western blotting detection of IL‐7R in TD‐EVs‐Normal and TD‐EVs‐Colitis. (g) ELISA quantification of LPS levels in serum and thymus. (h) LPS administration via tail vein injection in mice, LPS levels in serum and thymus were quantified using ELISA. (i) qRT‐PCR analysis of IL‐7R expression post‐LPS treatment. (j) Immunofluorescence of thymic IL‐7R post‐LPS treatment. (k) TD‐EVs‐LPS was analyzed using negative‐staining TEM and western blotting detection of TSG101, CD9, CD81, IL‐7R levels. (l) *Ex vivo* imaging of DiR‐labeled TD‐EVs‐Colitis and TD‐EVs‐LPS in colon, spleen, liver, femur, tibia, brain, thymus, and lung. (m) Neutrophils were incubated with PKH26‐labeled TD‐EVs‐Normal, TD‐EVs‐Colitis, TD‐EVs‐LPS, and EVs internalization was examined using laser scanning confocal microscopy. (n–q) NET formation assay: Neutrophils were treated with PBS, TD‐EVs‐Normal, TD‐EVs‐Colitis, TD‐EVs‐LPS, or PMA, with/without anti‐IL‐7R antibody. NETs were quantified. TD‐EVs‐Normal: EVs from thymus of normal mice; TD‐EVs‐Colitis: EVs from thymus of DSS‐induced colitis mice; TD‐EVs‐LPS: EVs from thymus of LPS‐treated mice; IL‐7R (‐): anti‐IL‐7R antibody. *n* = 4–5 mice per group; results are presented as the mean ± SD; *p < 0.05, **p < 0.01, ***p < 0.001, ****p < 0.0001; ns: non‐significance.

IL‐7R is essential for T cell development, differentiation, survival, and maintenance [23], and is also detectable in plasma [29]. The thymus serves as the primary site of T cell development, differentiation, and maturation. We measured IL‐7R expression levels in the colon, thymus, bone marrow, brain, liver, and spleen. IL‐7R expression was predominantly detected in the thymus and was significantly higher in colitis mice compared to controls (Figure 8a,b; Figure S12a). To directly determine the contribution of the thymus to circulating IL‐7R‐enriched EVs, we established a DSS‐induced colitis model in athymic Nude mice. We isolated serum EVs from Normal (C57BL/6), Colitis (C57BL/6), and Colitis (Nude) mice and analyzed IL‐7R levels in serum EVs by nanoflow cytometry. The results demonstrated that IL‐7R expression in serum EVs was increased in colitis (C57BL/6) mice relative to Normal (C57BL/6) mice. However, athymic Nude colitic mice exhibited lower IL‐7R levels in serum EVs than C57BL/6 colitic mice (Figure 8c). Furthermore, analysis of human thymic tissues revealed IL‐7R expression, with significantly higher levels in thymoma lesions compared to peritumoral thymic tissue (Figure S13). Thymus‐derived EVs (TD‐EVs) were isolated and characterized (Figure 8d; Figure S14), revealing that the upregulated IL‐7R in the thymus of colitis mice could be packaged into thymus‐derived EVs (TD‐EVs‐Colitis) (Figure 8e,f). Furthermore, IL‐7R levels in EVs isolated from the colon, thymus, bone marrow, brain, liver, and spleen of mice were compared. TD‐EVs‐Colitis showed significant enrichment of IL‐7R compared with EVs derived from other organs (Figure S15).

Lipopolysaccharide (LPS), is a known inducer of IL‐7R upregulation [30]. We observed markedly elevated LPS levels in both serum and thymus tissues of colitis mice, suggesting that LPS derived from the inflamed gut may reach the thymus via systemic circulation (Figure 8g). To explore whether thymic LPS exposure drives IL‐7R expression, we administered LPS via tail vein injection in mice (Figure 8h). Compared to other organs, LPS‐treated mice exhibited significant upregulation of IL‐7R specifically in the thymus (Figure 8i,j; Figure S12b). Thymus‐derived EVs from LPS‐treated mice were isolated and characterized (Figure 8k; Figure S14), and the elevated IL‐7R was confirmed to be packaged into thymus‐derived EVs (TD‐EVs‐LPS) (Figure 8k). Moreover, TD‐EVs‐LPS were significantly enriched in IL‐7R relative to EVs from other organs (Figure 8k; Figure S16). Through in vivo and in vitro experiments, we found that after blocking the LPS‐stimulated signal with anti‐TLR4 blocking antibody, the IL‐7R levels in thymus tissue, its EVs, and thymocytes were decreased (Figure S17). To track the biodistribution of TD‐EVs, we labeled TD‐EVs‐Colitis and TD‐EVs‐LPS with the fluorescent dye DiR and injected them intravenously. Results revealed that TD‐EVs‐Colitis and TD‐EVs‐LPS can accumulate in the colon (Figure 8l). Furthermore, we observed that TD‐EVs‐Colitis and TD‐EVs‐LPS were internalized by neutrophils (Figure 8m). TD‐EVs‐Colitis and TD‐EVs‐LPS robustly induced NET formation and synergistically enhanced PMA‐triggered NETosis (Figure 8n–q; Figure S18). Importantly, neutralizing IL‐7R with a specific antibody significantly reduced the NET‐inducing capacity of both TD‐EVs‐Colitis and TD‐EVs‐LPS (Figure 8n–q; Figure S18).

Collectively, these findings indicate that the thymus is the primary source of circulating IL‐7R‐enriched EVs, whose production is amplified by LPS stimulation during colitis. These EVs migrate via the thymus‐gut axis to the colon, where they drive NET formation and exacerbate intestinal inflammation.

## 3 Discussion

IBD is a systemic disorder characterized by complex interactions between the gut and extraintestinal organs, which synergistically promote disease progression. However, its etiology remains poorly understood, and there are no effective curative treatments, with frequent relapses. In this study, we demonstrated that LPS generated during colitis enters the bloodstream and reaches the thymus, stimulating the expression of IL‐7R. Thymus‐derived IL‐7R‐enriched EVs are subsequently released into circulation and transported via the thymus‐gut axis to the colon, where they induce NET formation, exacerbating intestinal inflammation. These findings suggest that circulating IL‐7R‐enriched EVs may serve as novel blood‐based biomarkers for IBD diagnosis and therapeutic intervention.

Current IBD treatments primarily include anti‐inflammatory agents, immunosuppressants, and TNF blockers, such as 5‐aminosalicylic acid (5‐ASA), steroids, antimicrobials, and monoclonal antibodies targeting TNF‐α (e.g., infliximab, IFX). For mild to moderate UC, 5‐ASA remains the first‐line therapy for induction and maintenance of remission. Moderate to severe UC often requires corticosteroids for rapid symptom control, followed by biologics (e.g., anti‐TNF agents, vedolizumab, ustekinumab) or small‐molecule inhibitors (e.g., tofacitinib, ozanimod) to sustain remission. Despite therapeutic advances, clinical trials report response rates of only 30%–60%, and up to 20% of UC patients require hospitalization within five years of diagnosis, with 7% undergoing colectomy [31]. Similarly, Crohn's disease management relies on risk stratification, patient preference, and anti‐TNF‐α therapies, yet long‐term remission remains elusive for both UC and Crohn's disease [32]. A critical limitation of current therapies may lie in their exclusive focus on local gut pathology, overlooking the contributions of extraintestinal organs.

The thymus, a central immune organ responsible for T‐cell development and maturation, emerged in our study as a key extraintestinal player in colitis. We revealed that IL‐7R‐enriched EVs derived from the thymus migrate via the thymus‐gut axis to the colon, where they drive NET formation and aggravate inflammation. This highlights the thymus as a previously unrecognized contributor to IBD pathogenesis. According to statistics by James D. Lewis and colleagues, the age distribution of IBD onset is concentrated between 10 and 29 years, with UC showing higher incidence in the 20–29 age group and CD in the 10–19 age group [14]. The peak incidence of IBD coincides with the period of rapid thymic involution, suggesting a close association between thymic changes and IBD onset. Our findings also provide some explanation for why IBD predominantly affects children and young adults.

EVs in IBD have been implicated in amplifying inflammation [33]. For instance, EVs from IBD patient plasma activate STING signaling in macrophages, mirroring effects of LPS‐damaged epithelial cell‐derived EVs [34]. Our findings extend this paradigm by showing that blocking EV secretion in colitis models reduces IL‐7R expression, neutrophil infiltration, and NET formation, ultimately ameliorating disease severity.

Neutrophils are important players in intestinal innate immunity, and their infiltration into the intestinal mucosa is a hallmark of active IBD [35]. However, the role and functionality of neutrophils in IBD have received relatively less attention than other immune cells like T cells [35]. Emerging evidence suggests that neutrophils exhibit various phenotypes and functionalities under normal and pathological conditions [36]. As primary defenders against microbial invasion, a substantial accumulation of neutrophils in the intestinal mucosa causes damage to the epithelial barrier [37]. Once recruited, neutrophils exacerbate inflammation through the release of reactive oxygen species, cytotoxic granules, and NETs [38, 39]. Several studies suggested increased NET production in IBD [40]. Elevated levels of proteins associated with NETs were observed in samples from patients with UC [41]. The histones within NETs have been shown to compromise the integrity and permeability of the intestinal epithelial barrier, implicating their detrimental impact [42]. Additionally, the accumulation of NETs in both colon tissue and plasma significantly heightens the risk of venous thromboembolism in individuals suffering from active IBD [43]. The exosome‐mediated transfer of LINC00668 exacerbates thrombosis by facilitating the formation of NETs in the context of IBD [44]. Our study links circulating IL‐7R‐enriched EVs to NET induction, suggesting a mechanistic bridge between thymic signaling and neutrophil‐mediated pathology. Mechanistically, we demonstrate that IL‐7R induces NET formation via the PAD4 pathway to aggravate colitis.

Under normal physiological conditions, IL‐7R plays a fundamental role in lymphopoiesis and immune homeostasis. It is essential for T cell development and, in mice, for B cell development, as well as for the differentiation, survival, and maintenance of naïve and memory T cells [23]. IL‐7R expression persists on both naïve and memory T cells, and signaling through this receptor is critical for the long‐term maintenance of all T cell populations, primarily by promoting cell survival through modulation of the intrinsic apoptosis pathway [45]. In contrast, while mature B cells do not express IL‐7R, the development of pre‐pro‐B cells and subsequent early B cell maturation in mice is strictly IL‐7‐dependent [46]. Furthermore, IL‐7R is required for the development and maintenance of innate lymphoid cells (ILCs), thereby supporting the generation of lymphoid structures and barrier defense [23]. Evidence also indicates a role for IL‐7R in the development of fetus‐derived macrophages [47].

Beyond its physiological roles, IL‐7R is strongly implicated in the pathogenesis of various immune‐mediated diseases [23]. Elevated levels of soluble IL‐7R detected in the serum and urine of systemic lupus erythematosus (SLE) patients correlate with disease activity and anti‐dsDNA antibody concentrations [48, 49, 50]. Similarly, IL‐7R expression in monocytes from rheumatoid arthritis patients is associated with disease severity [51]. Studies have shown that collagen‐induced arthritis and bone erosion are alleviated by IL‐7R antibody therapy [52, 53]. In the context of IBD, IL‐7R signaling influences responsiveness to anti‐TNF therapy and T‐cell migration to the gut [24]. Blocking IL‐7R has been shown to suppress both adaptive and innate inflammatory responses in experimental models of colitis [54]. Our experimental modulation of IL‐7R in colitis models confirmed its pathogenic role: IL‐7R downregulation alleviated colitis, whereas its overexpression exacerbated inflammation.

The *IL7RA* gene undergoes alternative splicing, resulting in a soluble form of IL‐7R detectable in the plasma [55]. The presence of soluble IL‐7R in serum has been closely linked to lupus nephritis among patients with SLE [56]. We identified the thymus as the primary source of circulating IL‐7R‐enriched EVs in colitis. LPS from gut microbiota, entering circulation via damaged epithelium, upregulates thymic IL‐7R expression, which is packaged into EVs for systemic delivery. These EVs then facilitate NET‐driven colonic inflammation, establishing a feedforward loop between gut dysfunction and thymic signaling. It should be noted, however, that the involvement of the thymus‐gut axis in colitis progression demonstrated here is based on animal models. Further research is required to determine whether this axis operates in human IBD patients. Additionally, thymus‐specific IL‐7R‐deficient models would provide even more direct causal evidence of thymus‐originated IL‐7R and colitis.

In conclusion, our study unveils a novel thymus‐gut axis in IBD, mediated by IL‐7R‐enriched EVs that promote NET formation and exacerbate colitis. Targeting IL‐7R, EVs, or NETs may offer new therapeutic avenues.

## 4 Experimental Section

### 4.1 Mouse Models and Ethics

Male C57BL/6J mice, and Nude mice, aged 6 weeks, were acquired from the Guangdong Medical Laboratory Animal Center, China. *PAD4*‐KO mice were purchased from Cyagen Biosciences Inc (China). All experimental groups consisted of age‐ and sex‐matched animals. The care and handling of animals, along with all experimental protocols, adhered to the guidelines of the Animal Care and Use Committee of Guangzhou Medical University (G2023‐726). These guidelines are in alignment with the National Institute of Health's Guidelines for the Care and Use of Laboratory Animals in China.

### 4.2 Human Samples and Ethics

A diagnosis of UC and CD was established based on clinical, radiological, endoscopic, and histological criteria. Human samples were collected from the Second Affiliated Hospital of Guangzhou Medical University, following the informed consent of participants. All procedures involving human samples were approved by the Internal Review and Ethics Boards of the Second Affiliated Hospital of Guangzhou Medical University (KY‐EC2024‐017‐02).

### 4.3 DSS Colitis Induction and Treatment

Acute colitis was induced in the mice through the administration of 3% (w/v) DSS (36–50 kDa, MP Biomedicals) for a period of nine days (day 1 to day 9). The mice were divided into several groups for the study: Normal, DSS, DSS (Nude mice), DSS + GW4869, DSS + anti‐Ly‐6G antibody, DSS + DNase I, DSS + anti‐IL‐7R antibody (IL‐7R (‐)), DSS + Recombinant mouse IL‐7R (IL‐7R (+)), LPS, and LPS + anti‐TLR4 antibody. The intervention groups (DSS + GW4869, DSS + anti‐Ly‐6G, DSS + DNase I, DSS + IL‐7R (‐), DSS + IL‐7R (+), LPS, LPS + anti‐TLR4 antibody) received intraperitoneal injections of GW4869 (MCE, HY‐19363,2.5 mg/kg), anti‐Ly‐6G antibody (BioLegend, 127680, 200 µg per mouse), DNase I (Sigma–Aldrich, 10104159001, 20 mg/kg), anti‐IL‐7R antibody (Elabscience, E‐AB‐F10230, 20 µg per mouse), Recombinant mouse IL‐7R (MCE, HY‐P72536, 20 µg per mouse), LPS (intravenous injection via tail vein, Solarbio, L8880, 200 µg per mouse), and anti‐TLR4 antibody (intravenous injection via tail vein, BioLegend, 117617, 200 µg per mouse), respectively, on days 1, 3, 5, and 7. The Normal and DSS control groups were administered an equivalent volume of PBS via intraperitoneal injection.

### 4.4 Neutrophils Isolation

Mouse neutrophils were obtained from bone marrow cells of femurs and tibias. After creating a single‐cell suspension, neutrophils were separated through Percoll density gradient centrifugation and subsequent positive selection for CD11b^+^Ly‐6G^+^ cells using flow cytometry (BD Influx, USA). The isolated neutrophils were cultured at 37°C in a humidified atmosphere containing 5% CO_2_.

### 4.5 EVs Purification and Identification

EVs were isolated using differential centrifugation. Initially, human plasma samples underwent low‐speed centrifugation (700 ×*g*, 30 min, 4°C) in 15 mL polypropylene tubes using a swinging bucket rotor in a refrigerated centrifuge (Eppendorf 5804R, model A‐4‐44, Germany). The supernatants from this step were further centrifuged at 3500 ×*g* for 30 min at 4°C. Subsequently, the supernatants were transferred to 1.5 mL tubes and centrifuged at 20 000 ×*g* for 60 min at 4°C in a fixed angle rotor (model #3331, Thermo Electron Corporation, USA). The final centrifugation step involved ultracentrifugation of the supernatants at 120 000 ×*g* for 90 min at 4°C (Optima L‐100xp, Beckman Coulter, USA) in Quick‐Seal tubes. The resulting EV pellet was resuspended in PBS.

For identification, the EVs underwent negative‐staining TEM. A 3% phosphotungstic acid solution was used for staining, post which samples were examined with a FEI Tecnai G2 Spirit Twin TEM (FEI, USA). NTA was performed using a NanoSight NS300 (Malvern Instruments, UK) to characterize the EV particles. EVs identity and purity were verified by Western blotting analysis for characteristic marker proteins. Freshly isolated EV samples were lysed using RIPA buffer supplemented with phosphatase and protease inhibitors (Thermo Fisher Scientific, USA), and the protein concentration was quantified using a BCA protein assay kit (Beyotime, China). Equal amounts of EVs (30 µg) were separated on 10% SDS‐PAGE gels under denaturing conditions and subsequently transferred to PVDF membranes (GE Healthcare Life Sciences, UK). The membranes were blocked with 5% skim milk in TBST. After blocking, the membranes were incubated overnight at 4 °C with the following primary antibodies: TSG101 (Abcam, ab125011, 1:1000), CD81 (Abcam, ab109201, 1:1000), CD9 (Abcam, ab236630, 1:1000), and Calnexin (Proteintech, 81938‐1‐RR, 1:5000). Calnexin, an endoplasmic reticulum marker, was used as a negative control for EVs. After incubation with primary antibodies, the membranes were washed with TBST and then incubated with HRP‐conjugated secondary antibodies for 2 h at room temperature. Protein bands were visualized using an ECL detection system (Amersham, USA).

### 4.6 Neutrophils Treatment

For neutrophil treatment, cells were incubated on coverslips and treated as follows: plasma EVs from healthy volunteers (Normal‐EVs), ulcerative colitis patients (UC‐EVs), and Crohn's disease patients (CD‐EVs) at 20 µg/mL for 24 h; GW4869 (10 µM) for 24 h; PMA, 150 nM, a NET‐inducer, for 4 h; IL‐7R (20 µg/mL) for 24 h.

### 4.7 Thymocytes Treatment

The thymus of normal mice was ground to obtain mouse thymocytes. For thymocyte stimulation, cells were incubated in culture dishes and treated as follows: LPS (100 ng/mL) for 24 h; Anti‐TLR4 antibody (2.5 µg/mL) for 24 h.

### 4.8 Analysis of EVs Uptake

Equal amounts of EVs (200 µg) were labeled with PKH26 (Sigma–Aldrich, USA) for 5 min before stopping the staining with an equal volume of 1% BSA. The EVs were then centrifuged at 120 000 ×*g* for 90 min at 4°C (Optima L‐100xp ultracentrifuge, Beckman Coulter) to pellet the PKH26‐labeled EVs, which were subsequently resuspended in PBS and centrifuged again under the same conditions. Neutrophils were incubated with these labeled EVs for 1 h, followed by confocal microscopy to assess EV internalization. Actin filaments and nuclei were visualized using Alexa Fluor phalloidin (CST) and DAPI, respectively.

### 4.9 Immunofluorescence Analysis

Cells were fixed with 4% paraformaldehyde for 25 min at room temperature, permeabilized with 0.1% Triton X‐100 in PBS for 10 min, and then blocked in PBS with 2% BSA for 30 min at room temperature. Following that, cells were incubated overnight at 4°C with anti‐MPO (Abcam, ab300650, 1:100) and anti‐H3cit (CST, 97272, 1:400) antibodies. Colon tissues were fixed, embedded in paraffin, and sectioned. Sections were deparaffinized, rehydrated, and blocked with 1% BSA. The sections were incubated overnight at 4°C with anti‐CD15 (Abcam, ab115993, 1:100), anti‐CD66b (Proteintech, 30875‐1‐AP, 1:100), anti‐Ly‐6G (CST, 88876, 1:100), anti‐CD11b (Abcam, ab184308, 1:100), anti‐MPO (Abcam, ab300650, 1:100), anti‐H3cit (CST, 97272, 1:400), anti‐IL‐7 (Abcam, ab175380, 1:100), and anti‐IL‐7R (Abcam, ab118527, 1:100) antibodies. The cells and sections were subsequently incubated with the appropriate Alexa Fluor‐conjugated secondary antibodies. Nuclei were detected using SYTOX Green (ThermoFisher Scientific, S7020, 1:10000) and DAPI. Throughout the process, samples were washed three times in PBS for 5 min each. Visualization of the sections was performed using an LSM 800 laser scanning confocal microscope (Zeiss, Germany).

### 4.10 In vivo Biodistribution of EVs

For real‐time monitoring of extracellular vesicle trafficking in mice, DiR lipophilic membrane dye (UElandy, China) was utilized to fluorescently label purified EVs. Briefly, the labeling procedure involved incubating EV suspensions with 5 µM DiR at 37°C under gentle agitation for 30 min, adhering to the manufacturer's protocol. To remove unincorporated dye molecules, the labeled mixture was purified through ultracentrifugation (120 000 ×*g*, 4°C, 90 min) and subsequently reconstituted in 200 µL sterile PBS to generate DiR‐conjugated EVs (DiR‐EVs). In this study, DiR‐EVs were derived from different sources: DiR‐DSS‐EVs were plasma EVs isolated from mice in the DSS group; DiR‐TD‐EVs‐Colitis were thymic EVs from DSS group mice; and DiR‐TD‐EVs‐LPS were thymic EVs from LPS group mice. Mice in the DSS group were induced by administering 3% (w/v) DSS for 9 days. Mice in the LPS group were induced via intraperitoneal injections of LPS (200 µg per mouse) on days 1, 3, 5, and 7, with EVs isolated on day 9. Mice received intravenous, intraperitoneal and intragastric injections of DiR‐EVs (150 µg per mouse). Following a 24h circulation period, the mice were humanely euthanized and major organs including colon, spleen, liver, femur, tibia, brain, thymus, and lung were surgically excised. Tissue‐specific accumulation of fluorescently tagged EVs was quantitatively assessed using the Odyssey CLx near‐infrared imaging platform (LI‐COR Biosciences, USA) with excitation/emission wavelengths set at 748/780 nm.

### 4.11 Disease Activity Index

Throughout the treatment, mice were evaluated daily for body weight changes, diarrhea, and bleeding. Fecal blood presence was assessed with a Hemoccult assay kit (Nanjing Jiancheng Bio‐engineering Institute, China). Body weight changes were calculated relative to the initial day. DAI were determined based on weight loss, stool consistency, and bleeding.

### 4.12 Macroscopic Assessment and Histologic Evaluation

The mice were sacrificed on day 9, and the length of their colons was measured. A blinded and unbiased observer assessed the macroscopic scores of the colons. The parameters evaluated in the macroscopic scores included hyperemia, wall thickening, ulceration, inflammation extension, and damage, which were described in our previous study [57]. The colons were preserved in a 4% paraformaldehyde solution, underwent routine processing, and were embedded in paraffin. Subsequently, paraffin‐embedded colon sections were prepared and stained with hematoxylin and eosin (H&E). In a blinded manner, the histopathological scores were analyzed as previously described [57]. The scoring was based on the extent of inflammation, infiltration of neutrophils and lymphohistiocytes, extent of crypt damage, formation of crypt abscesses, presence of submucosal edema, loss of goblet cells, and occurrence of reactive epithelial hyperplasia in the colonic lesions.

### 4.13 AB‐PAS Staining

Colon sections were stained with Periodic acid Schiff and Alcian blue stain (AB‐PAS). The number of goblet cells per crypt was calculated.

### 4.14 RNA Extraction and Quantitative Real‐Time PCR Assay

The quantification of mRNA expression was conducted using qRT‐PCR. Briefly, RNA was extracted from mouse tissues by lysing 100 mg of sample in Trizol reagent (Invitrogen, USA), following the manufacturer's recommended protocol. The concentration of extracted RNA was determined using a Nano Drop ND‐2000 spectrophotometer (Thermo Scientific, USA). Subsequently, the complementary DNA (cDNA) was synthesized from 1.0 µg of total RNA by utilizing oligo (dT) primers and a Thermo Scientific Revert Aid First Strand cDNA synthesis kit (Thermo Scientific), following the manufacturer's protocol. The expression levels of IL‐17A, TNF‐α, IL‐1β, TGF‐β1, iNOS, IRAK4, RIPK1, RIPK3, PI3K, mTOR, MAPK, PAD4, Agr‐1, IL‐7R, and GAPDH were evaluated using a SYBR Green Master Mix kit (Takara, Japan) and the primers outlined in Table S1. GAPDH served as the internal control, and the 2^−ΔΔCT^ method was used to assess the fold‐change in expression.

### 4.15 Immunohistochemistry

Immunohistochemistry was performed on formalin‐fixed, paraffin‐embedded colon sections to evaluate the expression of IL‐17A (Proteintech, 26163‐1‐AP, 1:200), TNF‐α (BOSTER, BA0131, 1:100), IL‐1β (Proteintech, 26048‐1‐AP, 1:100), TGF‐β (Proteintech, 81746‐2‐RR, 1:50), iNOS (Affinity, AF0199, 1:800), and Arg‐1 (Proteintech, 1600‐1‐AP, 1:400). After deparaffinization, rehydration, and antigen retrieval in citrate buffer (pH 6.0) via microwave heating, endogenous peroxidase activity was quenched with 3% H_2_O_2_. Sections were blocked with 1% BSA for 60 min and then incubated overnight at 4°C with the respective primary antibodies. After washing, sections were incubated with HRP‐conjugated secondary antibodies for 30 min at room temperature. Signal was developed using 3,3′‐diaminobenzidine (DAB; Dako, Denmark) as the chromogen. Sections were counterstained with hematoxylin.

### 4.16 Western Blotting

For protein analysis, colon tissues and EVs were lysed using RIPA buffer with added phosphatase and protease inhibitors (Thermo Fisher Scientific, USA). The lysates, along with plasma and EVs‐free plasma samples, were separated on a 10% SDS‐PAGE gel. Proteins were then transferred to PVDF membranes (GE Healthcare Life Sciences, UK), which were blocked with 5% skim milk. Membranes were incubated overnight at 4°C with primary antibodies against IL‐7R (1:100). This was followed by incubation with HRP‐conjugated anti‐IgG secondary antibodies for 2 h at room temperature. Detection was performed using the ECL system (Amersham, USA).

### 4.17 ELISA

LPS concentrations in serum and thymus were measured using a commercial ELISA kit (Elabscience, China) according to the manufacturer's instructions.

### 4.18 Nanoflow Cytometry Analysis

The isolated EVs were incubated with the corresponding flow antibodies at room temperature in the dark for 60 min, followed by filling the volume with PBS. Subsequently, the anti‐IL‐7R mixture was subjected to ultracentrifugation at 120 000 × *g* for 90 min, and the pellet was resuspended in 120 µL of PBS prior to flow cytometric analysis on the machine.

### 4.19 Statistical Analysis

Results are presented as mean ± SD. Statistical differences between two groups were determined using unpaired two‐sample t‐tests. For multiple comparisons involving more than two groups, one‐way ANOVA was employed. Whereas the Kruskal‐Wallis H test with Dunn's multiple comparison post hoc analysis was applied for non‐normally distributed data. *P*‐values < 0.05 were considered statistically significant. Immunofluorescence and immunohistochemistry images were quantified using ImageJ software (NIH, USA). Statistical analyses were conducted using GraphPad Prism 9.0 (GraphPad Software, USA).

## Author Contributions

Y.L., Y.L., and R.Y. contributed equally to this work. L.W., J.W., Y.L., and Y.Y. conceived and designed experiments. Y.L., L.W., Y.L., R.Y., Z.Z., J.W., D.L., J.S., Y.H., F.C., and H.D. performed the experiments. Y.L., L.W., Y.L., R.Y., Z.Z., L.Y., L.Z., P.P., X.W., and C.C. provided critical samples/materials and reagents/protocols. Y.L., L.W., Y.L., R.Y., J.W., D.L., Y.H., Z.L., Y.W., and S.Z. analyzed and interpreted data. L.W., J.W., Y.L., Y.Y., and S.L. wrote the paper. All authors critically read and approved the final version of the manuscript.

## Funding

This work was supported by the National Natural Science Foundation of China (No. 82570023), National Key Research and Development Program of China (No. 2024YFE0214800), Science and Technology Plan Project of Guangzhou (No. 2023A04J0559), the Department of Education of Guangdong Province (No. 2023ZDZX2048), the Natural Science Foundation of Guangdong Province (Nos. 2020A1515011573, 2023A1515220167, 2025A1515010419), Open Project of Guangzhou Medical University, the Guangzhou key laboratory for clinical rapid diagnosis and early warning of infectious diseases (No. 202102100003), Guangzhou Science and Technology Fund (No. 2024A03J0791), Guangdong Basic and Applied Basic Research Foundation (No. 2022A1515111102), and Guangzhou Science and Technology Plan Project (No. SL2022A04J00697).

## Conflicts of Interest

The authors declare no conflicts of interest.

## Supporting information




**Supporting File**: advs76418‐sup‐0001‐SuppMat.docx.

## Data Availability

The data that support the findings of this study are available from the corresponding author upon reasonable request.
