# Supplementary material for: IL‐7R‐Enriched Extracellular Vesicles From the Thymus Drive Colitis via Promoting Neutrophil Extracellular Trap Formation
Source: Adv Sci (Weinh). 2026 Jul 6:e20331. Online ahead of print. doi: 10.1002/advs.202520331 (PMC13335758; doi:10.1002/advs.202520331)
Supplement: Supplementary file 1 — Supporting File: advs76418‐sup‐0001‐SuppMat.docx. [file ADVS-9999-e20331-s001.docx]

**IL-7R-enriched extracellular vesicles from the thymus drive colitis via promoting neutrophil extracellular trap formation**

**Supplemental Figures**

**
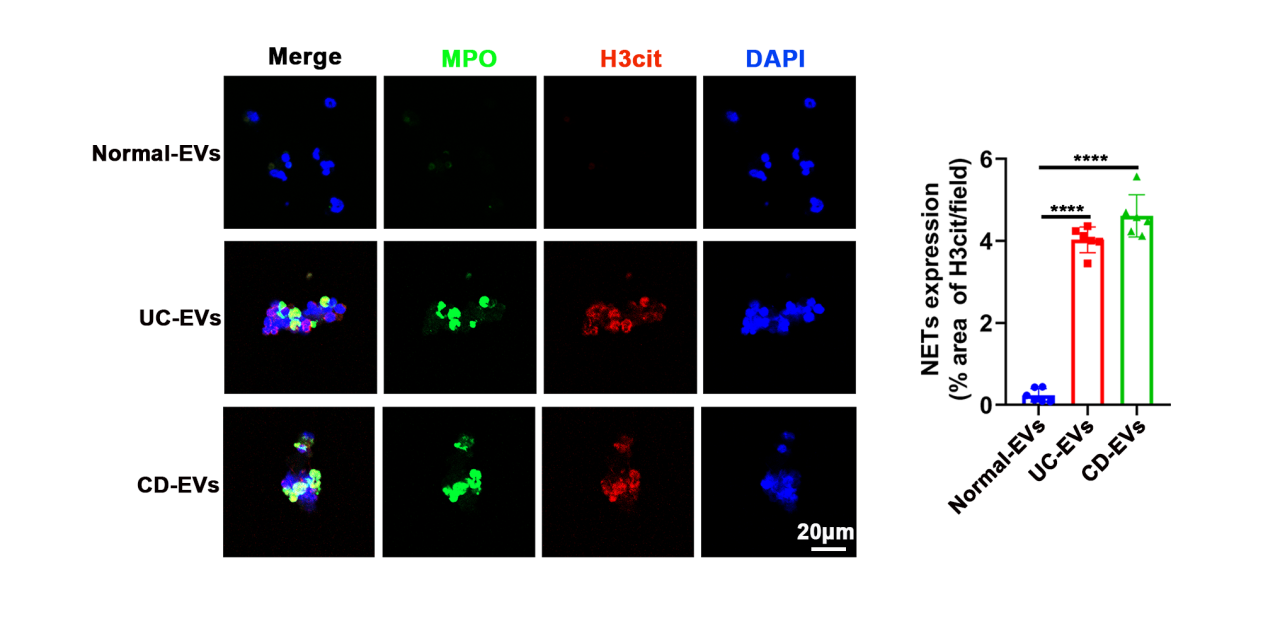
**

**Figure S1. Circulating extracellular vesicles from UC and CD patients induce NET formation.** Neutrophils were treated with Normal-EVs, UC-EVs, or CD-EVs. and NETs were observed based on H3cit and MPO co-localization. Results are presented as the mean ± SD; *****p* < 0.0001.


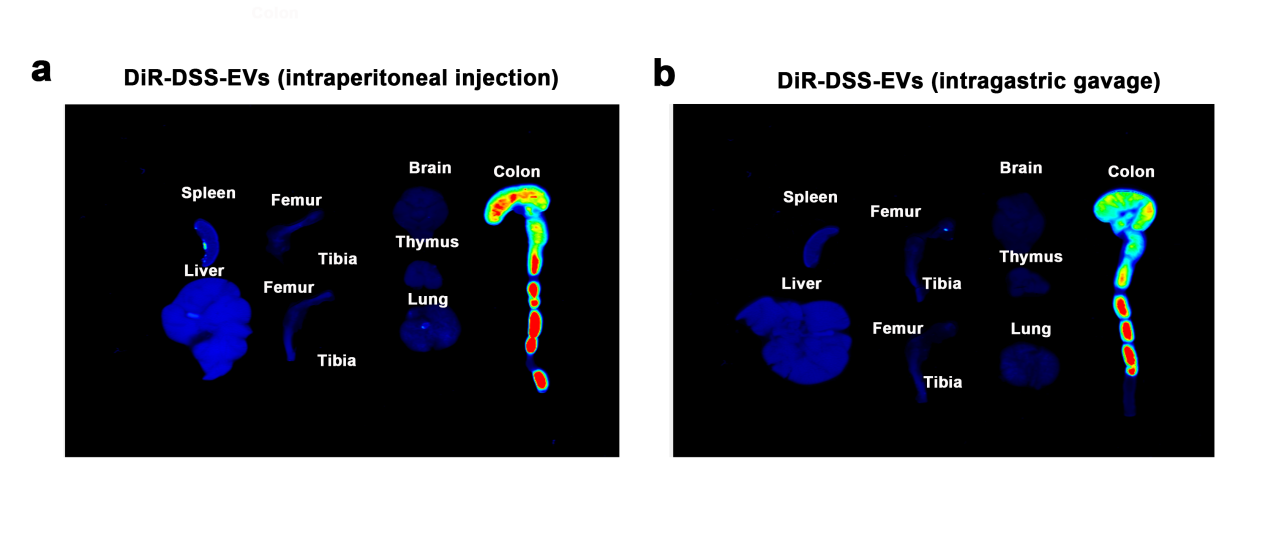


**Figure S2. DSS-EVs were consistently found to accumulate in the colon.** DiR-labeled DSS-EVs were separately administered to mice via intraperitoneal injection and intragastric gavage two routes to further verify the biodistribution pattern of EVs. DiR-labeled DSS-EVs delivered via these two routes were obviously enriched in the colon.

**
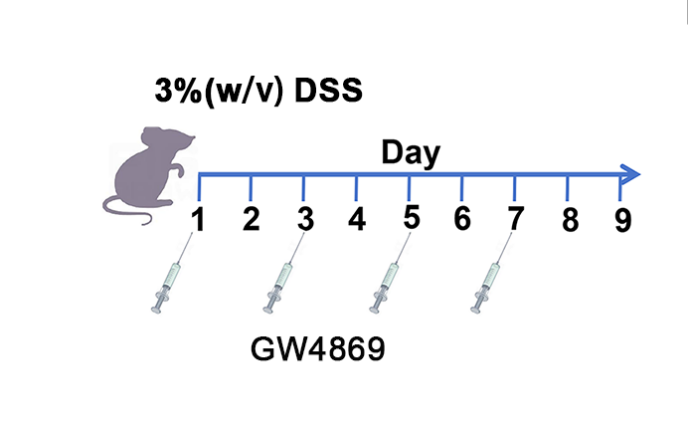
**

**Figure S3.** Intraperitoneal injections of GW4869 were administered on days 1, 3, 5, and 7 to inhibit EVs release in DSS-induced colitis mice.

**
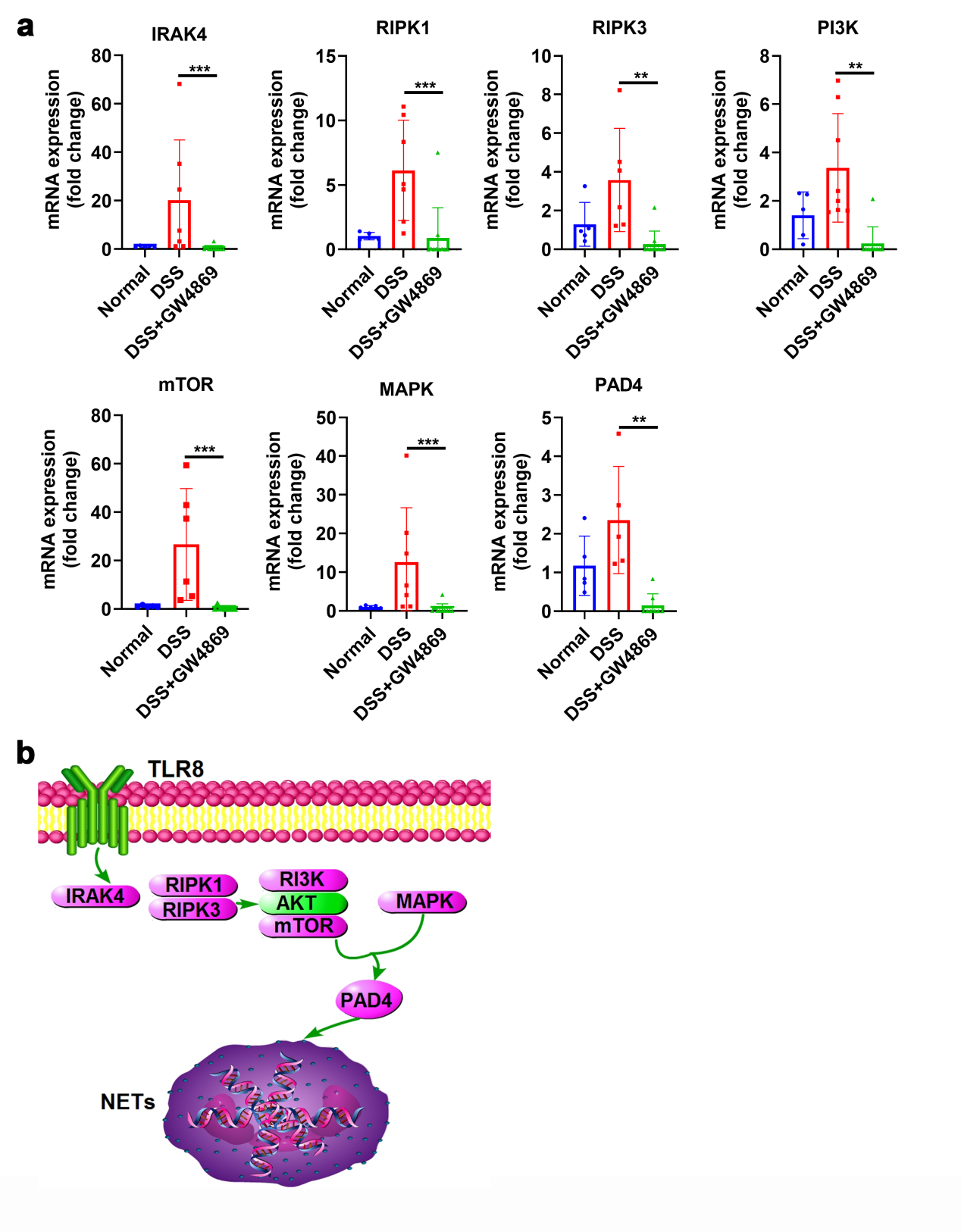
**

**Figure S4. Inhibiting EVs release notably decreased the expression of IRAK4, RIPK1, RIPK3, PI3K, mTOR, MAPK, and PAD4.** a) We administered intraperitoneal injections of GW4869 on days 1, 3, 5, and 7 to inhibit EVs release in DSS-induced colitis mice. On day nine, expressions of IRAK4, RIPK1, RIPK3, PI3K, mTOR, MAPK, and PAD4 in colon tissues were assessed via qRT-PCR. b) It was found that inhibiting EVs release impacts the crucial pathway in NETosis, reducing the expression of IRAK4, RIPK1, RIPK3, PI3K, mTOR, MAPK, and PAD4. n=5-9 mice per group; results are presented as the mean ± SD; ***p* < 0.01, ****p* < 0.001.


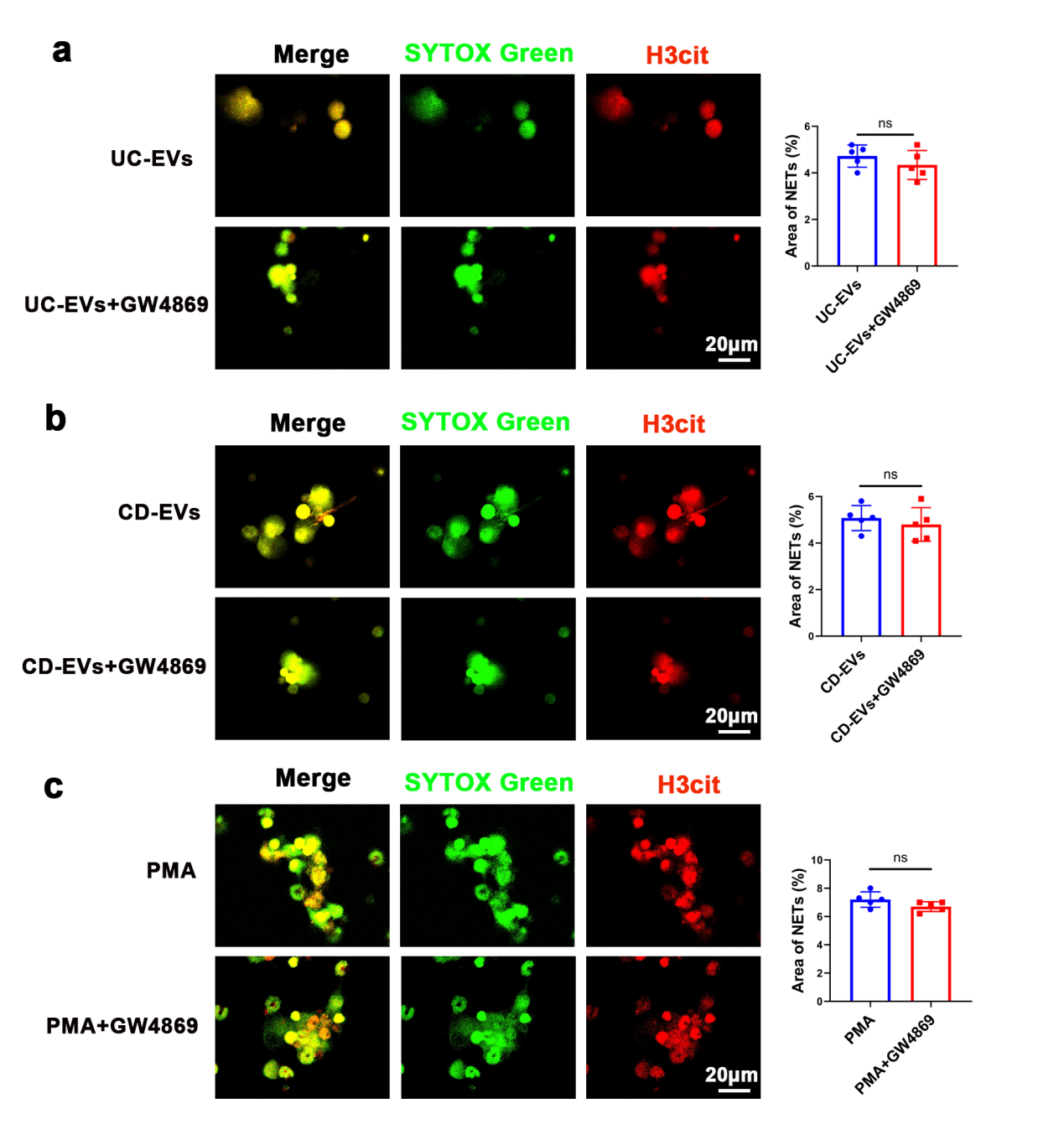


**Figure S5. GW4869 did not decrease NET formation induced by UC-EVs, CD-EVs, or PMA.** Neutrophils were stimulated with sufficient UC-EVs (a), CD-EVs (b) and PMA (c) in the presence of GW4869. NET formation was assessed by SYTOX Green/H3cit co-staining. Results are presented as the mean ± SD; ns: non-significance.


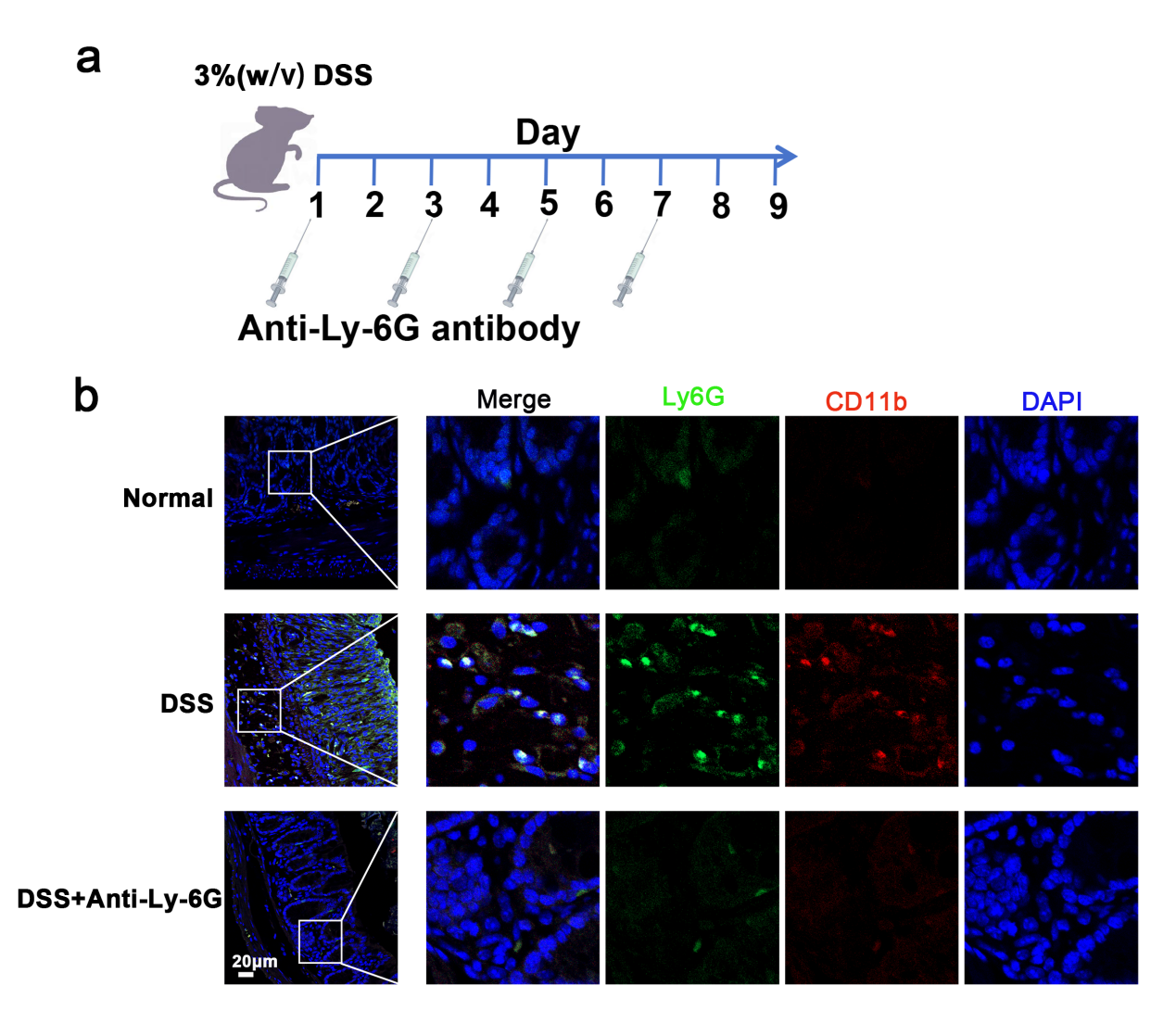


**Figure S6. Colon neutrophils in DSS-induced colitis mice were decreased with intraperitoneal injection of anti-Ly-6G antibody.** a) Intraperitoneal injections of anti-Ly-6G antibody were administered on days 1, 3, 5, and 7 to decrease the number of colon neutrophils in DSS-induced colitis mice. b) On day nine, the mice were sacrificed, and neutrophil levels in the colon tissues were quantified using immunofluorescence.


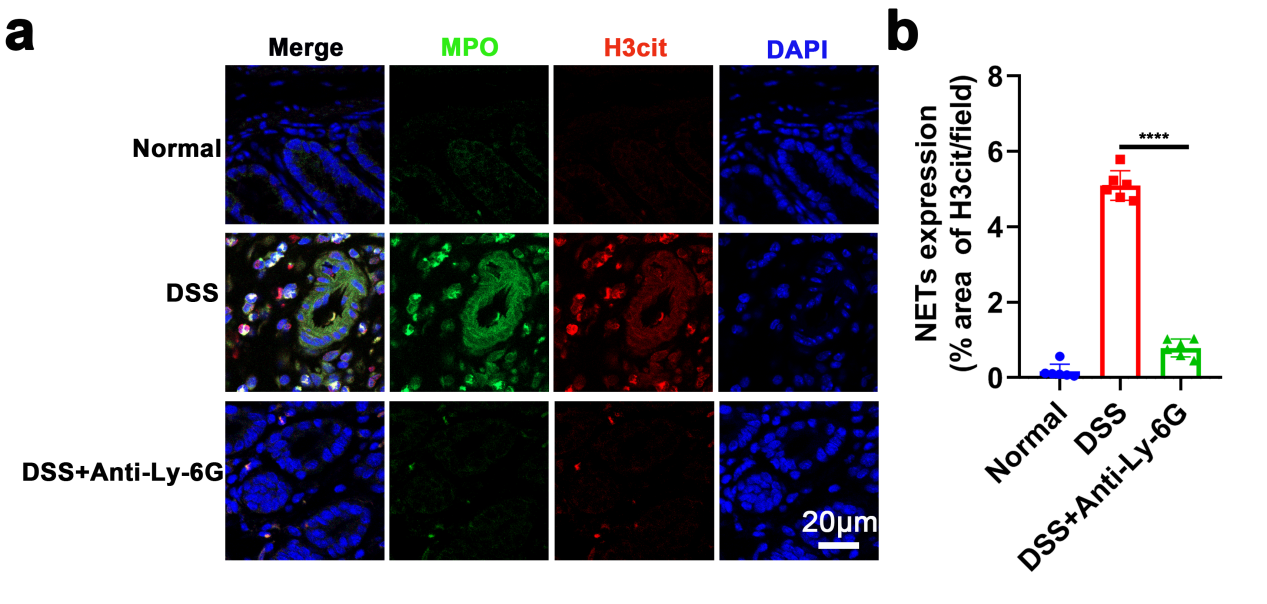


**Figure S7. Treatment with anti-Ly-6G antibody significantly reduced NET formation in the colon of DSS-induced colitis mice by decreasing colonic neutrophils.** a) Anti-Ly-6G antibody was administered intraperitoneally on days 1, 3, 5, and 7, leading to a reduction in colon neutrophils. On day nine, mice were sacrificed, and NET formation was assessed in colon tissues through immunofluorescence, identifying NETs by H3cit and MPO co-localization. b) A statistical analysis of NET formation followed. n=5-10 mice per group; results are presented as the mean ± SD; *****p* < 0.0001.


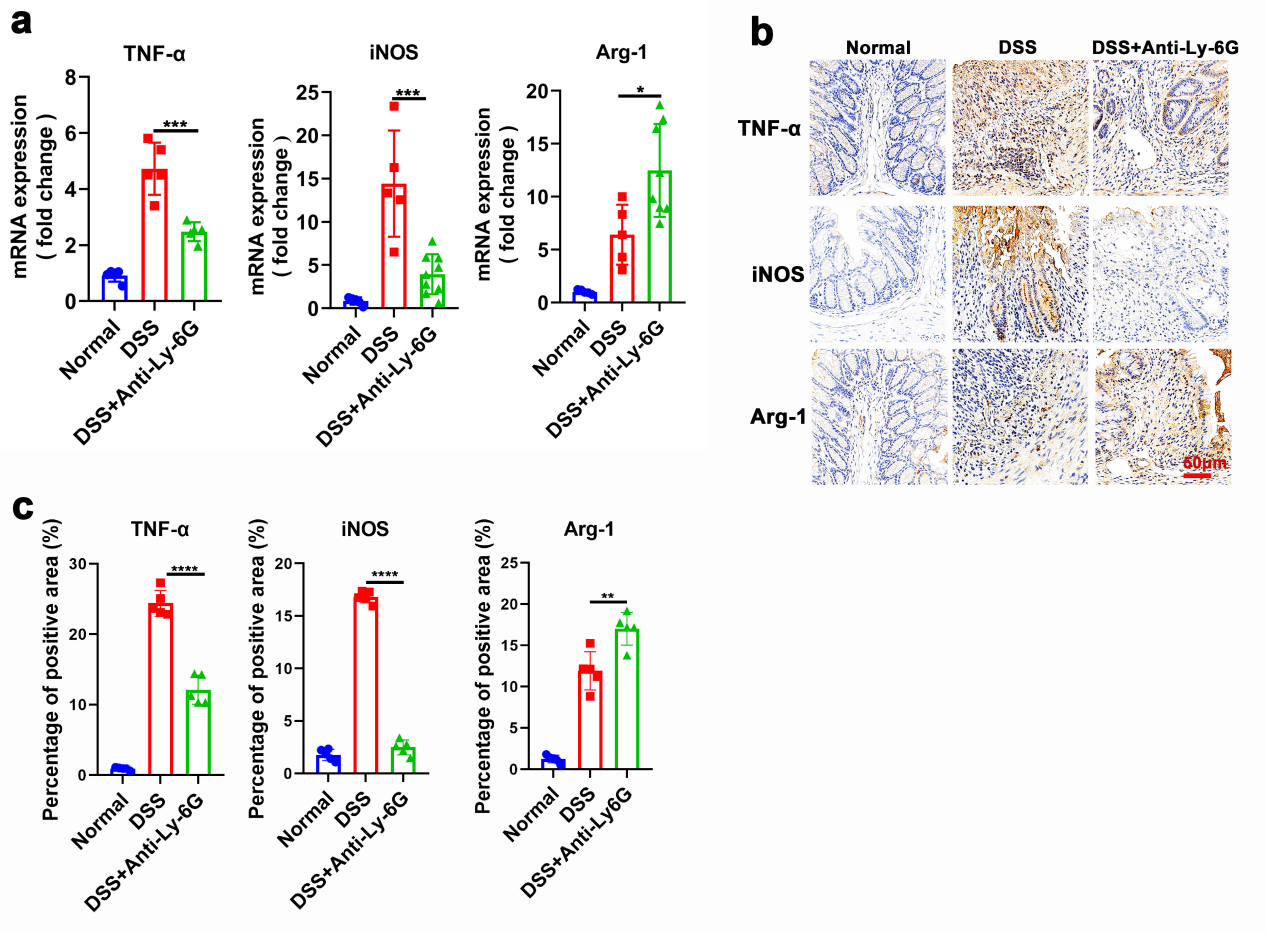


**Figure S8. Effect of neutrophil depletion on proinflammatory mediators.** a-c) Mice with DSS-induced colitis were treated with anti-Ly-6G antibody. On day 9, mRNA levels (a) and protein levels of TNF-α, iNOS, and Arg-1 (b, c) in colon tissues were measured by qRT-PCR and immunohistochemistry, respectively. n=5-10 mice per group; results are presented as the mean ± SD; **p* < 0.05, ***p* < 0.01, ****p* < 0.001, *****p* < 0.0001.

**
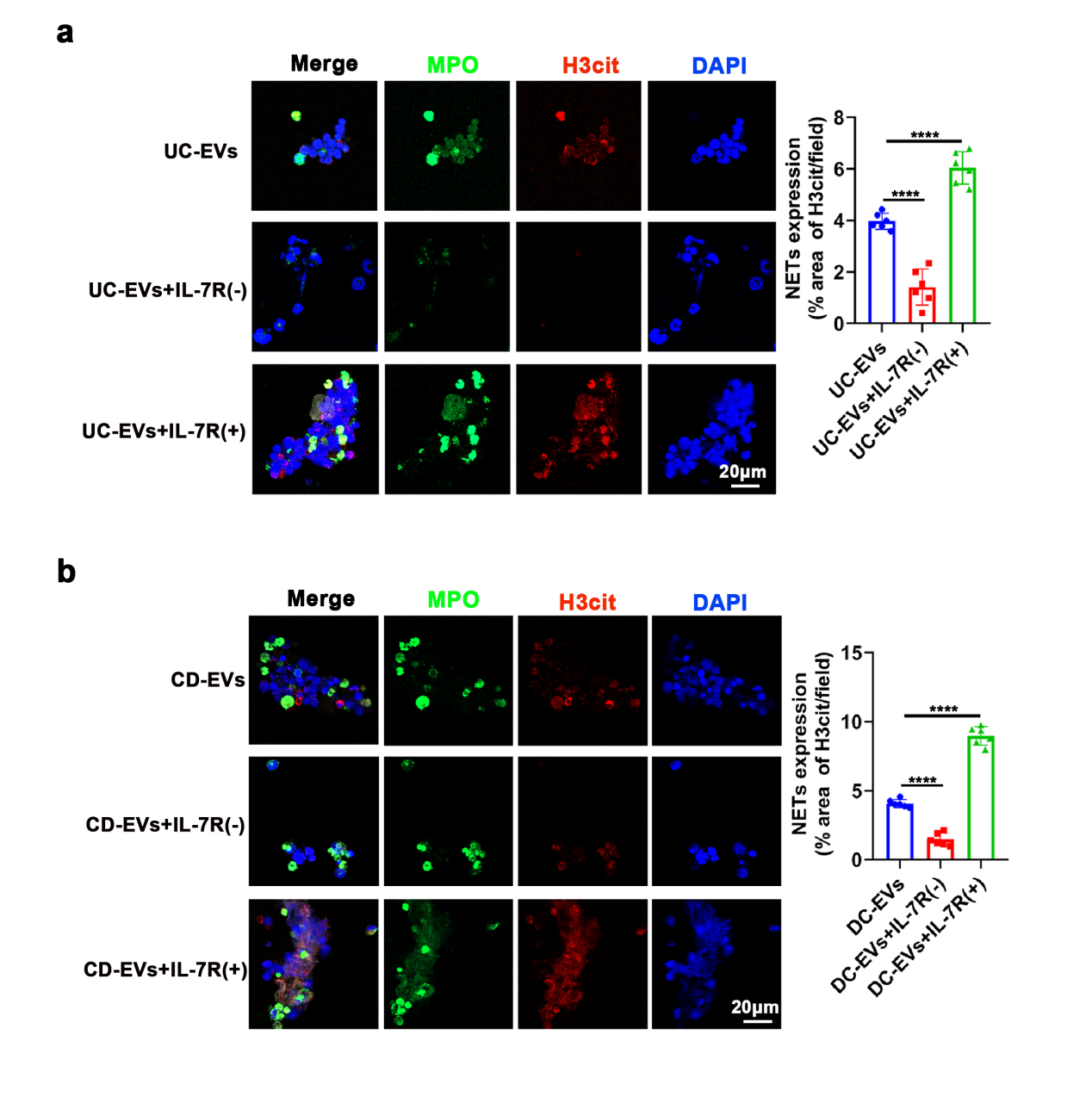
**

**Figure S9. Plasma-derived EVs of colitis are enriched with IL-7R and induce colonic NET formation through IL-7R.** a) Neutrophils were treated with UC-EVs, UC-EVs + anti-IL-7R antibody, or UC-EVs + recombinant mouse IL-7R protein, and NETs were detected based on H3cit and MPO co-localization. b) Neutrophils were treated with CD-EVs, CD-EVs + anti-IL-7R antibody, or CD-EVs + recombinant mouse IL-7R protein, and NETs were detected based on H3cit and MPO co-localization.Results are presented as the mean ± SD; *****p* < 0.0001.


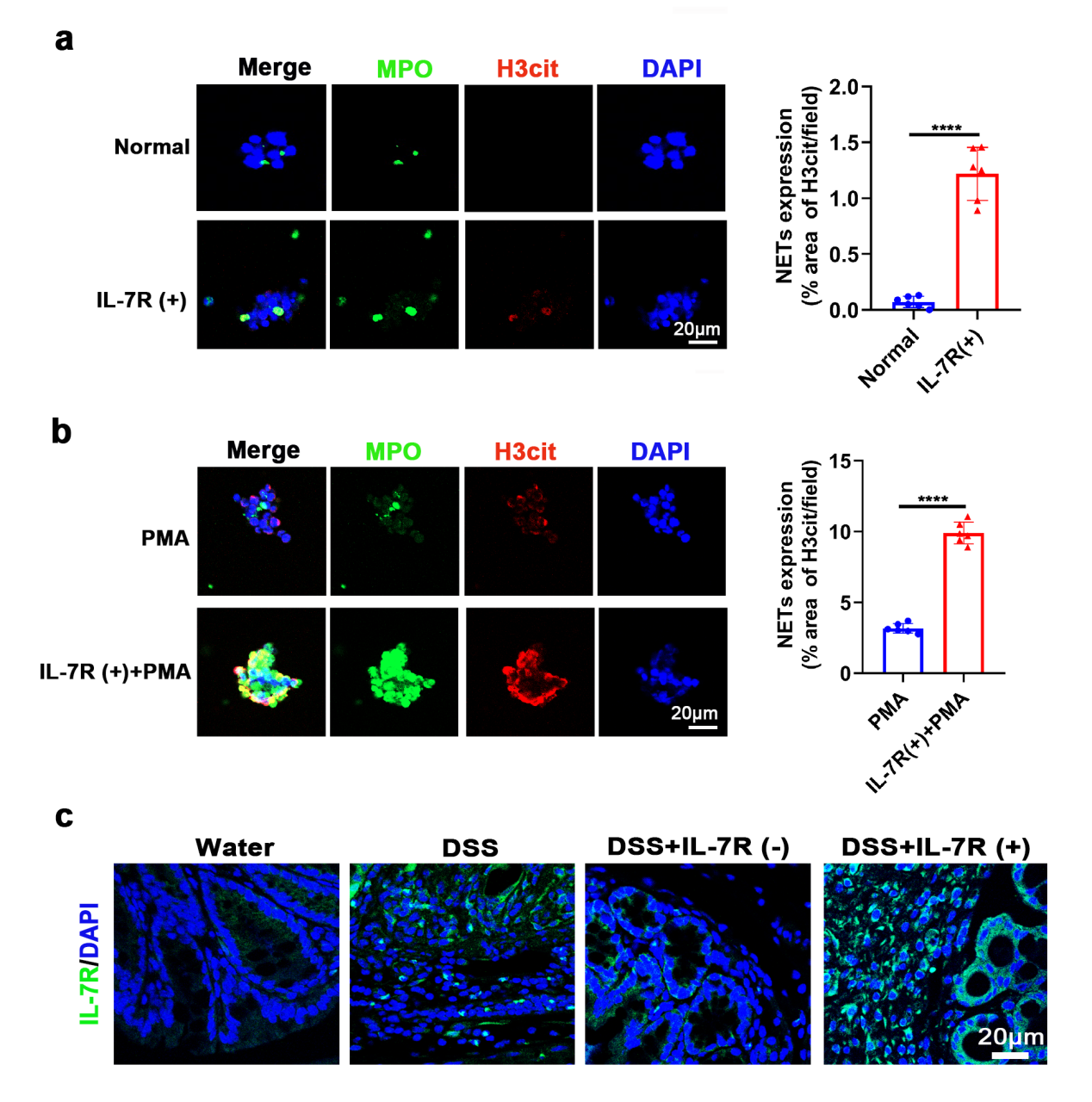


**Figure S10. IL-7R stimulation promoted NET formation.** a) Immunofluorescence analysis demonstrating that IL-7R promotes the formation of NETs. b) Immunofluorescence showing that IL-7R can promote the formation of PMA-induced NETs. c) We administered intraperitoneal injections of anti-IL-7R antibody (DSS+IL-7R (-)) and recombinant mouse IL-7R protein (DSS+IL-7R (+)) on days 1, 3, 5, and 7 to upregulate or reduce IL-7R expression in DSS-induced colitis mice. On day nine, the mice were sacrificed, and the level of IL-7R in the colon tissues was measured by immunofluorescence.Results are presented as the mean ± SD; *****p* < 0.0001.


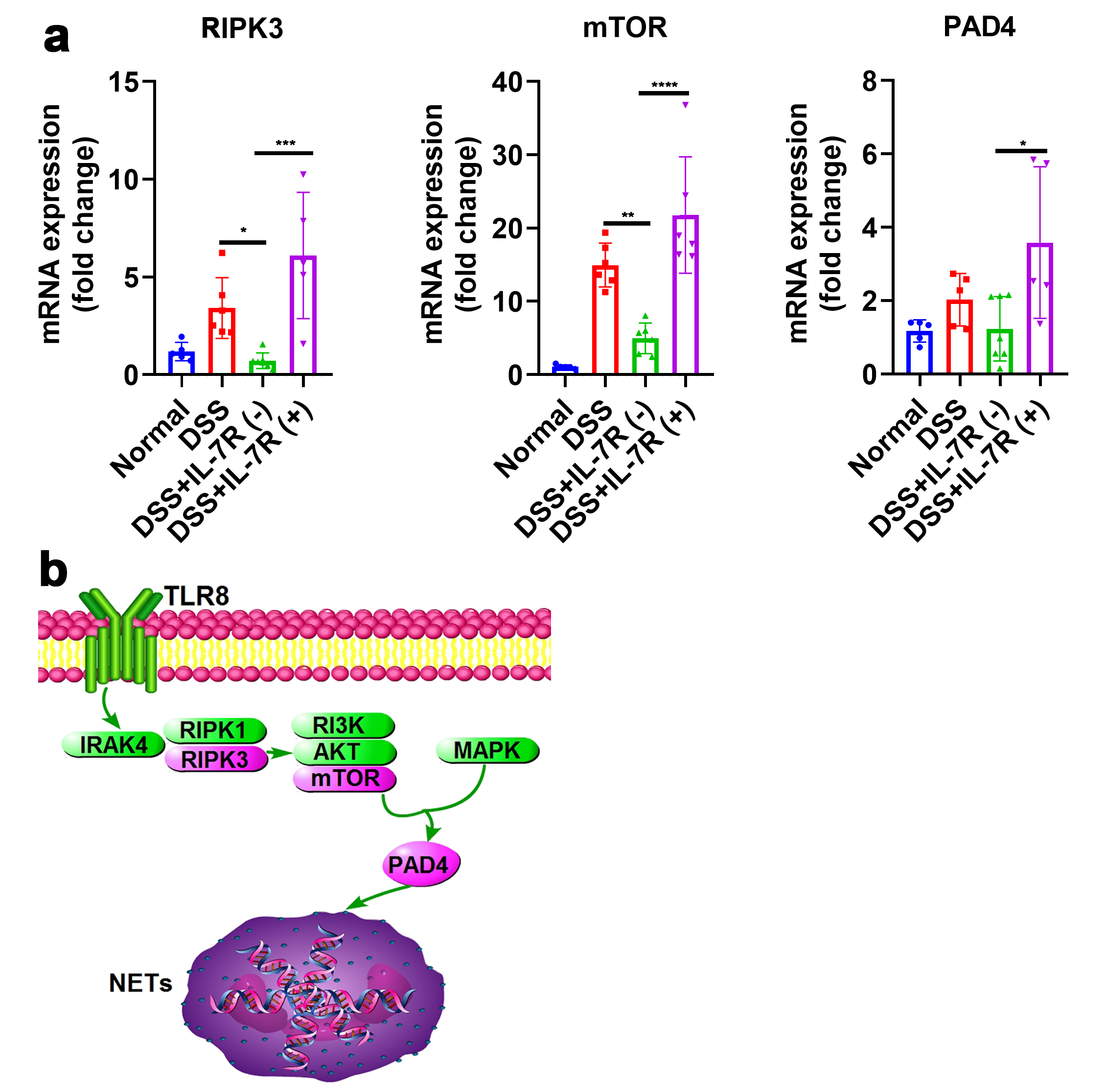


**Figure S11. Modulating IL-7R levels affected RIPK3, mTOR, and PAD4 expression.** a) To alter IL-7R levels, anti-IL-7R antibody (for reduction) and recombinant IL-7R protein (for elevation) were administered intraperitoneally on days 1, 3, 5, and 7 in DSS-induced colitis mice. On day nine, the expression levels of RIPK3, mTOR, and PAD4 in the colon were quantified via qRT-PCR. b) Molecular mechanisms regulating NETosis, deficiency of IL-7R reduced the expression levels of RIPK3, mTOR, and PAD4. n=5-10 mice per group; results are presented as the mean ± SD; **p* < 0.05, ***p* < 0.01, ****p* < 0.001, *****p* < 0.0001.


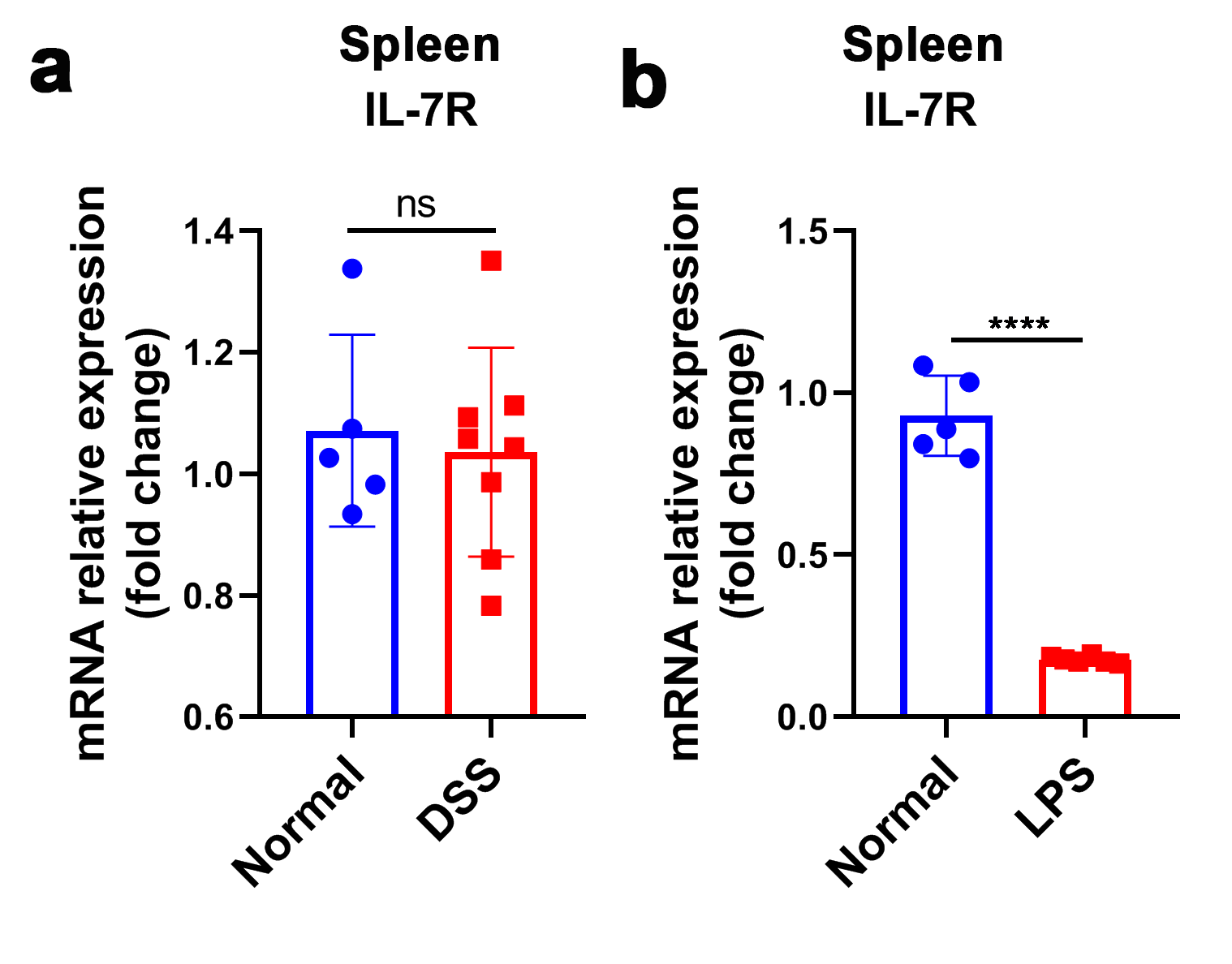


**Figure S12.** **qRT-PCR analysis of IL-7R mRNA expression in spleen tissues of mice.** a) qRT-PCR analysis of IL-7R expression in spleen tissues of DSS-induced colitis. b) qRT-PCR analysis of IL-7R expression in spleen tissues of mice after LPS treatment. n=5-10 mice per group; results are presented as the mean ± SD; *****p* < 0.0001; ns: non-significance.


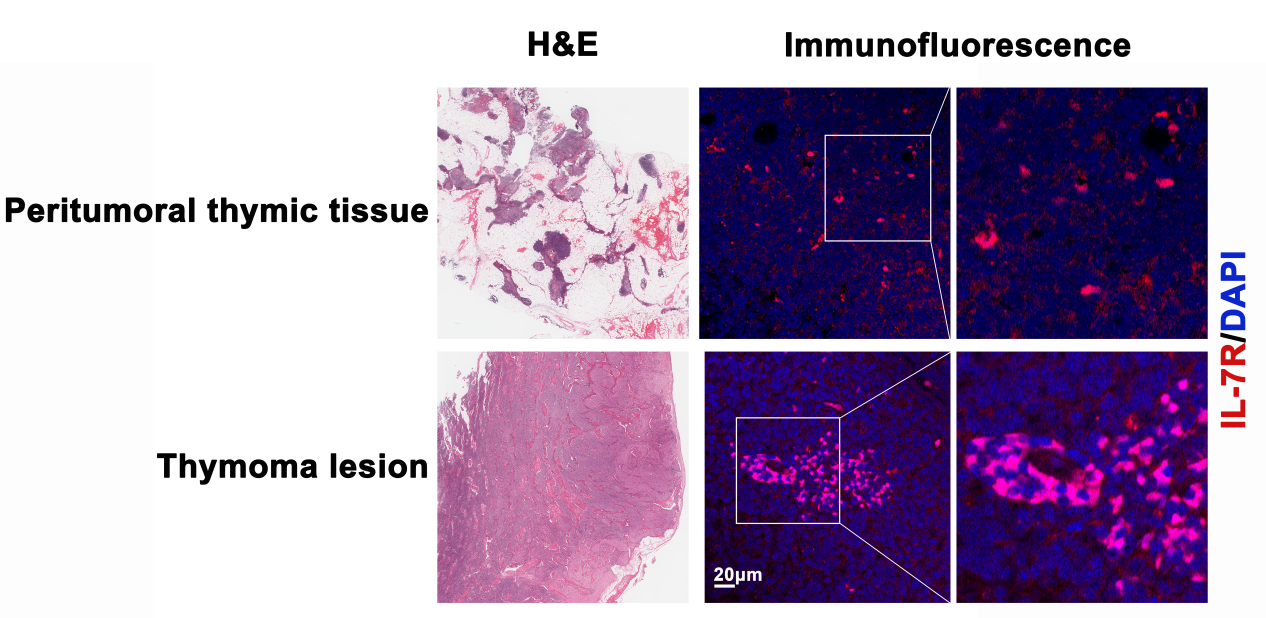


**Figure S13.** Histopathological changes in human peritumoral thymic tissue and thymoma lesions were assessed by H&E staining, while IL-7R expression was evaluated via immunofluorescence.


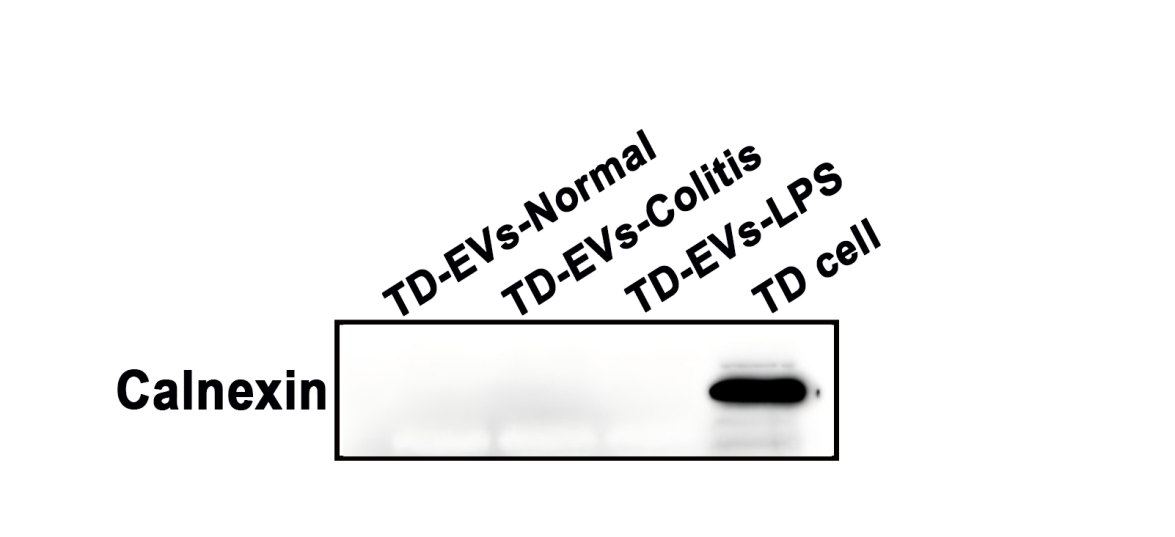


**Figure S14.** Western blotting analysis of protein levels of Calnexin in EVs lysates, with thymus-derived cells served as a positive control for the detection of Calnexin.


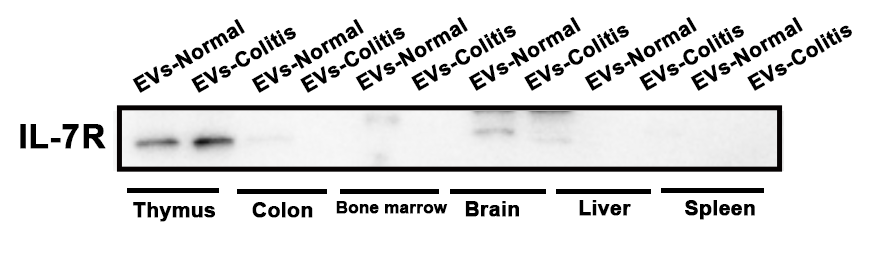


**Figure S15. TD-EVs-Colitis are enriched in IL-7R.** Western blot analysis of IL-7R levels in EVs isolated from the thymus, colon, bone marrow, brain, liver, and spleen of colitis mice.


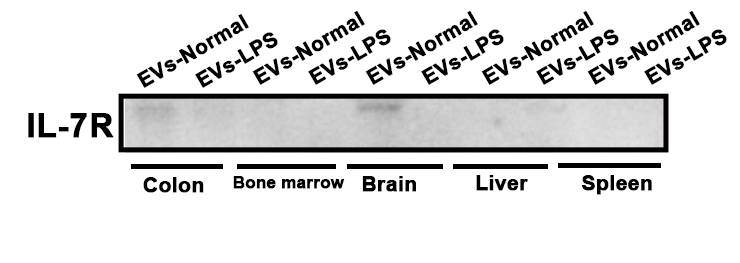


**Figure S16. TD-EVs-LPS are enriched in IL-7R.** Western blot analysis of IL-7R levels in EVs isolated from the colon, bone marrow, brain, liver, and spleen of LPS-treated mice.


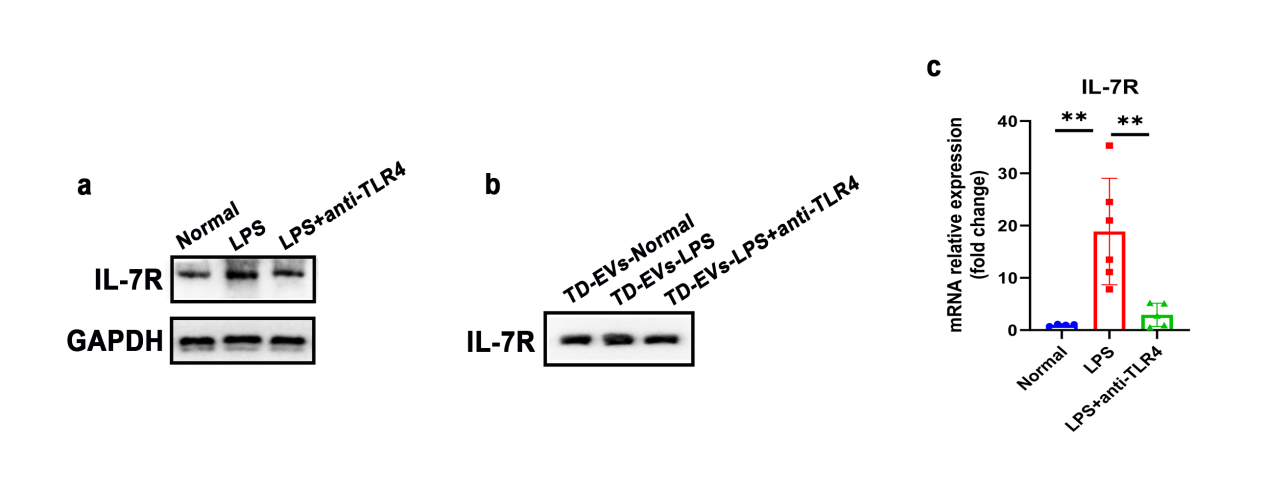


**Figure S17. After blocking the LPS-stimulated signal with anti-TLR4 blocking antibody, the IL-7R levels in both thymus tissue and its EVs were decreased.** a) Western blotting analysis of IL-7R expression in thymus tissues of LPS-challenged mice with or without anti-TLR4 treatment. b) Western blotting analysis of IL-7R expression in TD-EVs of LPS-challenged mice with or without anti-TLR4 treatment. c) qRT-PCR analysis of IL-7R expression in thymocytes following *in vitro* LPS and LPS+anti-TLR4 stimulation. Results are presented as the mean ± SD; ***p* < 0.01.


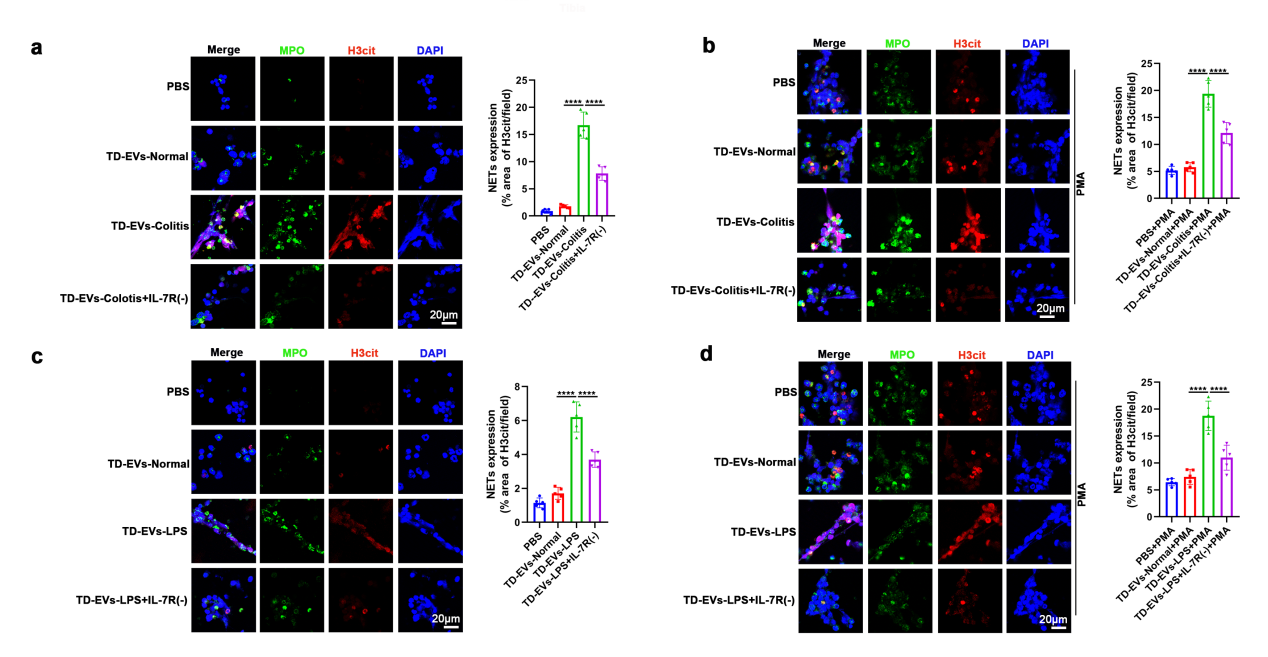


**Figure S18. TD-EVs-Colitis and TD-EVs-LPS robustly induced NET formation.** a-d) NET formation assay: Neutrophils were treated with PBS, TD-EVs-Normal, TD-EVs-Colitis, TD-EVs-LPS, or PMA, with/without anti-IL-7R antibody. NETs were quantified. TD-EVs-Normal: EVs from thymus of normal mice; TD-EVs-Colitis: EVs from thymus of DSS-induced colitis mice; TD-EVs-LPS: EVs from thymus of LPS-treated mice; IL-7R (-): anti-IL-7R antibody. n=4-5 mice per group; results are presented as the mean ± SD; *****p* < 0.0001.

**Table S1. Primers used for qRT-PCR**

| **Gene** | **Forward (5'-3')** | **Reverse (5'-3')** |
| --- | --- | --- |
| IL-17A | CAGACTACCTCAACCGTTCCAC | TCCAGCTTTCCCTCCGCATTGA |
| TNF-α | GGTGCCTATGTCTCAGCCTCTT | GCCATAGAACTGATGAGAGGGAG |
| IL-1β | TGGACCTTCCAGGATGAGGACA | GTTCATCTCGGAGCCTGTAGTG |
| TGF-β1 | TGATACGCCTGAGTGGCTGTCT | CACAAGAGCAGTGAGCGCTGAA |
| iNOS | GAGACAGGGAAGTCTGAAGCAC | CCAGCAGTAGTTGCTCCTCTTC |
| IRAK4 | GGTCCAGATTGAGCTGTTTGCC | GTTTGTGCCACTGTTGCCGCTT |
| RIPK1 | GACTGTGTACCCTTACCTCCGA | CACTGCGATCATTCTCGTCCTG |
| RIPK3 | GAAGACACGGCACTCCTTGGTA | CTTGAGGCAGTAGTTCTTGGTGG |
| PI3K | ACCATCAGTGGCTCTGCGGTTT | GTGGTCTTCTGGGAACTCACCT |
| mTOR | AGAAGGGTCTCCAAGGACGACT | GCAGGACACAAAGGCAGCATTG |
| MAPK | TCAAGCCTTCCAACCTCCTGCT | AGCTCTGTACCAACGTGTGGCT |
| PAD4 | ACGCTGCCTGTGGTCTTTGACT | ACCTCCAGGTTCCCAAAGGCAT |
| IL-7R | CACAGCCAGTTGGAAGTGGATG | GGCATTTCACTCGTAAAAGAGCC |
| Arg-1 | TGTCCCTAATGACAGCTCCTT | GCATCCACCCAAATGACACAT |
| GAPDH | ACTCCACTCACGGCAAATTC | TCTCCATGGTGGTGAAGACA |
